# Oropouche virus as an emerging cause of acute febrile illness in Colombia

**DOI:** 10.1080/22221751.2022.2136536

**Published:** 2022-10-14

**Authors:** Karl A. Ciuoderis, Michael G. Berg, Lester J. Perez, Abbas Hadji, Laura S. Perez-Restrepo, Leidi Carvajal Aristizabal, Kenn Forberg, Julie Yamaguchi, Andres Cardona, Sonja Weiss, Xiaoxing Qiu, Juan Pablo Hernandez-Ortiz, Francisco Averhoff, Gavin A. Cloherty, Jorge E. Osorio

**Affiliations:** a Global Health Institute One-Health Colombia, Universidad Nacional de Colombia, Medellín, Colombia; b Infectious Diseases Research, Abbott Diagnostics, Abbott Park, IL, USA; c Global Health Institute, University of Wisconsin, Madison, WI, USA; d Abbott Pandemic Defense Coalition, Chicago, IL, USA

**Keywords:** Oropouche, bunyavirus, acute febrile illness, fever, Colombia, RT–PCR, NGS, serology

## Abstract

Arbovirus infections are frequent causes of acute febrile illness (AFI) in tropical countries. We conducted health facility-based AFI surveillance at four sites in Colombia (Cucuta, Cali, Villavicencio, Leticia) during 2019-2022. Demographic, clinical and risk factor data were collected from persons with AFI that consented to participate in the study (*n* = 2,967). Serologic specimens were obtained and tested for multiple pathogens by RT–PCR and rapid test (Antigen/IgM), with 20.7% identified as dengue positive from combined testing. Oropouche virus (OROV) was initially detected in serum by metagenomic next-generation sequencing (mNGS) and virus target capture in a patient from Cúcuta. Three additional infections from Leticia were confirmed by conventional PCR, sequenced, and isolated in tissue culture. Phylogenetic analysis determined there have been at least two independent OROV introductions into Colombia. To assess OROV spread, a RT-qPCR dual-target assay was developed which identified 87/791 (10.9%) viremic cases in AFI specimens from Cali (3/53), Cucuta (3/19), Villavicencio (38/566), and Leticia (43/153). In parallel, an automated anti-nucleocapsid antibody assay detected IgM in 27/503 (5.4%) and IgG in 92/568 (16.2%) patients screened, for which 24/68 (35.3%) of PCR positives had antibodies. Dengue was found primarily in people aged <18 years and linked to several clinical manifestations (weakness, skin rash and petechiae), whereas Oropouche cases were associated with the location, climate phase, and odynophagia symptom. Our results confirm OROV as an emerging pathogen and recommend increased surveillance to determine its burden as a cause of AFI in Colombia.

## GRAPHICAL ABSTRACT

**Figure:**
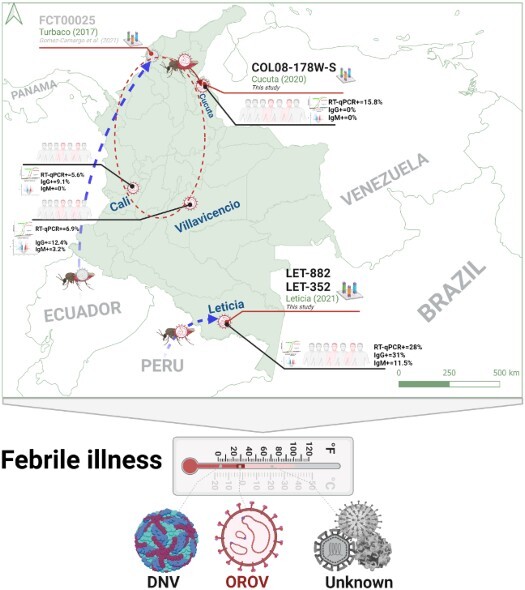

## Introduction

Arboviruses are endemic to the tropical regions of the Americas and frequently result in large outbreaks. Oropouche virus (OROV) is an emerging arthropod-vectored virus from the genus orthobunyavirus and was first isolated in 1955 from an infected individual in Vega de Oropouche, Trinidad & Tobago [1]. Like other *Peribunyaviridae* family members, its viral genome consists of three negative-sense RNA segments that encode the RdRp (L), the viral glycoproteins (M), and the nucleocapsid (S). Over the last six decades, Oropouche fever has been identified in several countries in South and Central America and been implicated in >30 epidemics causing more than 500,000 cases [1,2]. OROV is second only to dengue as a leading cause of acute febrile illness in the region [2]. The infection typically presents as a self-limiting febrile illness that is clinically indistinguishable from other arbovirus infections with a variety of non-specific symptoms, including headache, arthralgia, myalgia, skin rash, chills, dizziness, photophobia, nausea, and vomiting [3]. The clinical course usually resolves within 7 days, but in some circumstances can progress to meningitis or encephalitis [1,4].

OROV infection is commonly found in the tropical climates of the Amazon basin and Central-Plateau regions but has also been seen in numerous countries throughout Latin America where environmental conditions are favourable for transmission [1]. In the urban cycle, the biting midge, *Culicoides paraensis*, and *Culex quinquefasciatus* mosquitoes serve as vectors, transmitting from infected persons possessing high titres of circulating OROV during the symptomatic phase [5]. In the sylvatic cycle (non-human transmission), the mosquito vector is still unclear, but the virus has been isolated from sylvatic mosquitoes such as *Ochlerotatus serratus* and *Coquillettidia venezuelensis.* Wild mammals including the sloth bear (*Bradypus tridactiyus*), non-human primates (*Aloutta sanguinus*), and various rodents and wild birds are important primary hosts for the sylvatic cycle. Direct human to human transmission has not been documented.

The actual burden of OROV in South and Central America is unknown, as routine surveillance is lacking and point of care tests are not commercially available. Studies carried out in Peru and Bolivia have reported that 2–6% of patients with undifferentiated fevers were infected with OROV [6, 7]. Reports of OROV isolated from febrile patients in French Guiana, Haiti, and Ecuador indicate its range is expanding [8-11]. In Colombia, OROV was first reported in 2017 from a 28-year-old woman harbouring a strain highly related to those found in neighbouring Ecuador in 2016 [12]. In this study, we conducted fever surveillance in four unique locations in Colombia and report OROV as a significant cause of undifferentiated acute febrile illness.

## Materials and methods

### Study area and population

**Figure 1.**
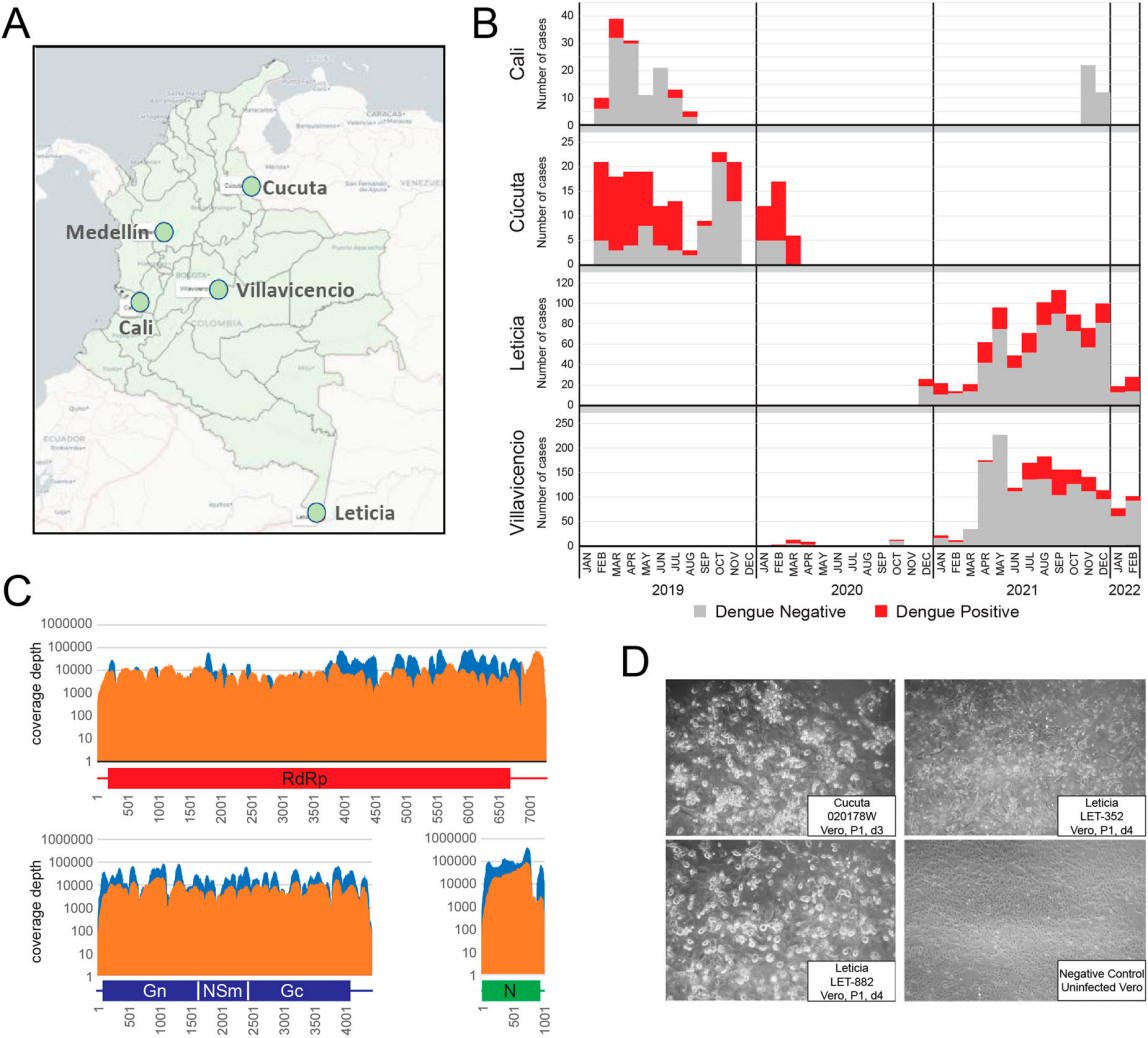
AFI surveillance and detection of Oropouche. A. Map of Colombia showing the four AFI surveillance sites and the city of Medellín where the testing laboratory is located. B. AFI pre-screening results by site, indicating the number of positive Dengue cases and those without a diagnosis. C. mNGS (orange) and CVRP (blue) coverage plots for L, M, and S genome segments of patient 0200178W. D. Oropouche virus was isolated on Vero cells by infecting sera from three individuals.

Health facility-based febrile illness surveillance was conducted in two sites in Colombia (Cali and Cucuta) from 2019 to March 2020. Two new study sites (Villavicencio and Leticia) were added in April 2020 at the same time surveillance was discontinued in the Cali and Cucuta sites. We report on the results from these four sites from 2019 through January 2022 (Figure 1(a)). Each facility provides health care services to cities/regions with populations ranging from 50,000 to 2,500,000 residents.

### Study design

Patients age 5 years and older who received outpatient care or were admitted with acute febrile illness (AFI) in the collaborating health facility, were asked to participate and enrolled in the study. AFI was defined as a measured temperature of ≥38.5°C using digital tympanic thermometers at the time of presentation or by self-reported history of fever in the preceding seven days. After obtaining informed consent, a study questionnaire was administered by a trained researcher to collect demographic, clinical, and epidemiologic/risk factor data. A venous whole blood sample was collected and tested by point of care rapid diagnostic tests for dengue (SD Bioline Dengue Duo) and malaria (Bioline Malaria Ag P.f/Pan). An additional sample was collected in a serum separator tube and centrifuged. The serum was then aliquoted into 2 ml tubes and stored at −80°C. Specimens were batched and transported at regular intervals under temperature-controlled conditions to the One Health Genomic Lab (Universidad Nacional, Medellin) for additional testing. Data collected on the questionnaire were de-identified and linked by a unique study ID number to the laboratory results and specimens. All data were transcribed into an electronic database. Source documents were scanned and compared to the electronic data for quality assurance. Ninety-four AFI samples that tested negative for known pathogens (Dengue, Zika, Chikungunya, Malaria) were initially sent in 2020 to Abbott Laboratories for metagenomic sequencing. An additional 791 AFI samples were sent to Abbott in 2021 for OROV screening.

### Ethics statement

The protocol for this study was approved by the ethics committee of Corporacion Investigaciones Biologicas (SC-6230-1), Hospital Erasmo Meoz (2018-136-009,224-2), Red de Salud Ladera (11-CEH/1127-19) and Hospital Departamental de Villavicencio (RDS-2020-064/104). Written informed consent was obtained from each participant. For participants age 17 and younger, minor assent was obtained and the parents or legal guardians provided written informed consent on their behalf. Samples and questionnaire data were anonymized prior to analysis.

### Multiplex Zika, chikungunya, and dengue (ZCD) real time RT–PCR

Dengue, Zika and chikungunya viruses were detected using a multiplex real time RT–PCR assay (ZCD) following a previously described protocol [13,14]. Additional details are available in the Online Supplement and Table S1.

### NGS sequencing

NGS sequencing was conducted at both Abbott Laboratories, Abbott Park, IL USA and Nacional University, Medellin, Colombia. Random-primed libraries derived from plasma specimens were sequenced by metagenomic and target enrichment approaches as described [15]. Metagenomic libraries were sequenced on a Novaseq at Novagene, Inc. (Sacramento, CA) and “target enriched” using Twist Bioscience’s Comprehensive Viral Research Panel (CVRP) probes followed by sequencing on a MiSeq (Illumina, CA, USA). NGS data was analyzed by SURPI and an Abbott-internal pipeline (data not shown) [16]. NGS data was deposited into SRA under BioProject PRJNA870952 and sequences were submitted to GenBank under accessions OP244877-OP244885.

### Conventional OROV RT–PCR

Conventional RT–PCR assay was conducted following a previously described protocol for the detection of the Nucleocapsid gene of OROV [17]. Additional details are available in the Online Supplement and Table S1.

### Cell culture isolation, plaque assay and virus titration

For virus isolation, OROV positive serum samples were inoculated in Vero cells and mosquito-derived C6/36 cells as described elsewhere [11]. To determine the infectious titre, plaque forming unit (PFU) assay was performed as described previously [18,19].

### Sequence dataset selection and phylogenetic analysis

All available full-genome OROV sequences (segments L, M and S) were downloaded from GenBank and merged into a multiple sequence alignment (Table S2) as described previously [20]. Genetic divergence between isolates was determined using Maximum Likelihood inference [21]. Genetic distances between the isolate obtained in the current study and the first strain of OROV in Colombia were estimated using MEGA X software [12,20,22].

### OROV RT-qPCR primer and probe design

Based on phylogenetic tree topology and lower genetic diversification (Figures S1,S2), target regions within genome segments L and M were selected for pan-Oropouche bunyavirus amplification. Primers and probes were designed using Oligo 7.6 software (Molecular Biology Insights, United States) as described, with selection parameters set to optimize priming efficiency in a TaqMan assay [23,24]. *In silico* evaluations for specificity using GenBank nt database and BLASTn searches were performed [24] and thermodynamic properties, secondary structures, and primer–primer interactions were evaluated using the OligoExplorer v1.2 and the OligoAnalyzer v1.1.2 software programs (Gene LinkTM, United States). Selected primers/probe sets are listed in Table S3.

#### Nucleic acid isolation and in vitro transcripts

Nucleic acid was extracted on the automated m2000sp platform using the TNA + Proteinase K protocol (Abbott Molecular, Des Plaines, IL). Targeted regions were cloned into the pBlueScript plasmid (Figure S3), linearized with *HindIII*, and RNA was generated with the MEGAscript kit (Ambion, Austin, TX).

#### OROV RT-qPCR, limit of quantification, efficiency, and analytical sensitivity

RT-qPCR experiments were performed on an m2000rt Real-Time PCR System instrument (Abbott Molecular, Des Plaines, IL, United States) and results analyzed in MultiAnalyze software. Reaction conditions and thermal profiles are listed in the Online Supplement and in Table S4. Serial dilutions of *in vitro* transcripts in water and plasma were detected by the respective primer/probe sets to determine linearity and efficiency (Figure S4). The limit of quantification (LoQ) was defined using ten-fold serial dilutions in nuclease-free water of quantified transcripts standards (Figure S4) [25]. Similarly, the analytical sensitivity (ASe) was estimated by evaluating ten-fold serial dilutions of quantified RNA transcripts standards, determined using a Probit regression analysis implemented in the MedCalc Statistical Software version 19.0.7 (MedCalc Software bvba, Ostend, Belgium; 2019). The ASe was defined as the concentration (copies of the transcripts/reaction) that yielded 95% positive results along with a 95% Confidence Interval (CI) [25].

#### RNA secondary structure

RNA for M and L segments were evaluated to avoid regions of secondary structure (Figure S5) [26]. The folding of RNA secondary structures was computed based on minimum free energy (MFE), equilibrium base-pairing probabilities, and the partition function parameters, using the RNAfold program included in the Vienna RNA software package version 2.0.0 (Institute for Theoretical Chemistry, University of Vienna, Austria) [27].

### OROV serology assay design

Available Oropouche nucleocapsid (N) sequences were aligned to derive a consensus sequence. Codons were optimized for expression of the full-length protein in *E. coli*. A C-terminal Histidine (His)-tag was fused to the ORF and cloned into pD444-CH (Atum, Newark, CA). Details of recombinant protein expression and purification are in the Online Supplement. The prototype ARCHITECT (Abbott, USA) Oropouche IgG or IgM assays are automated 2-step chemiluminescent microparticle immunoassays (CMIA) used for the qualitative determination of IgG or IgM antibodies to Oropouche. Cutoff settings were determined with 500 serum/plasma specimens from US (Figure S6). Western blots (WB) of Oropouche-infected Vero cell lysate were performed as described [28]. Competitive inhibition of anti-ORO-N binding to WB strips was performed by pre-incubation of the selected human samples with Oropouche recombinant nucleoprotein at room temperature for 30 min.

### Data analysis

A confirmed dengue case was defined as a RT–PCR positive result, a probable dengue case was defined as a NS1 RDT positive result, while a presumptive dengue case was defined as a negative result for RT–PCR or NS1 but positive for IgM antibody by RDT [29]. A confirmed Oropouche case was defined as a RT–PCR positive result, a presumptive Oropouche case was defined as a negative result for RT–PCR but positive for IgM antibody [30]. Confirmed, probable and presumptive OROV and DENV cases were included in the analysis as a positive case, while patients who did not fall into any category above were defined as a negative case. Past infection for OROV was defined as a positive result for IgG antibody. The index case plus the 791 samples screened by RT-qPCR were analyzed (*n* = 792).

Descriptive statistics were used to summarize the study data. Categorical variables were expressed as a percentage with 95% CI. Mean with standard deviation (SD), median with Interquartile ranges (IQR) were calculated for continuous variables or proportions for binary or categorical variables. Categorical data were cross-tabulated and quantitative variables were categorized. Tabular methods were used to distribute the number of observations per category.

To identify factors associated to DENV and OROV positive cases we fit a Generalized Linear model (GLM) using multivariate logistic regressions to estimate the Odds Ratio (OR) and their respective 95% CI. The DENV infection status (or Oropouche) was defined as the dependent variable. Clinical, epidemiological, or biological relevant variables, including potential confounders were considered in the final adjusted model. Model fit and selection were conducted using the Akaike Information Criterion (AIC). Variance inflation factor (VIF) was also calculated to assess multicollinearity of variables in the best-fitted model. Plausible interactions terms among the variables in the best-fitted model were evaluated by automatic stepwise process. The likelihood ratio test (*p*-value ≤ 0.1) was used to identify a significant effect.

## Results

### Acute cases of OROV detected in Colombia

Passive surveillance of undifferentiated acute febrile illness in four selected health facilities in Colombia was conducted from February 2019 to March 2020 in the cities of Cali and Cúcuta and from February 2020 to January 2022 in Leticia and Villavicencio (Figure 1(a,b)). We identified 2,967 patients that presented with AFI. The majority of AFI samples assessed were collected during 2021 (2358; 79.5%). Participants ranged in age from 5 to 89 years with a mean of 32.7 years (IQR = 19; Median = 30), and 55.7% (*n* = 1654) were female. Of the total samples tested, 5.5% (*n* = 164) were from Cali, 6.5% (*n* = 193) from Cucuta, 58.1% (*n* = 1,723) from Villavicencio, and 29.9% (*n* = 887) were from Leticia.

Dengue was detected in 615 out of 2,967 samples (20.7%) either by RT–PCR or rapid test (antigen/IgM), and all specimens tested negative for Mayaro, Chikungunya and Zika virus. There were 17/164 (10.3%) dengue cases detected in Cali, 112/193 (58%) in Cúcuta, 268/1723 (15%) in Villavicencio, and 218/887 (24%) in Leticia (Figure 1(b)). From 206 febrile cases tested for SARS-CoV-2 by RT-qPCR there were 27 positives, while for 309 cases screened by rapid test for malaria, only 3 were positive. Therefore, roughly 78% of AFI cases did not have an infectious agent identified to explain their illness.

Ninety-four non-dengue AFI specimens in total from any site were randomly selected for metagenomic next-generation sequencing. Using both unbiased, metagenomic NGS and CVRP virus target enrichment approaches, we detected OROV bunyavirus reads in the serum of a 25-year-old male patient (0200178W) from Cucuta (Figure 1(c)). Full length sequences for all three genome segments were obtained by both methods, each with coverage depths >1000X. The patient did not reported travel outside the municipality during the 15 days before symptom onset.

OROV infection was confirmed by conventional, one-step RT–PCR (Supplemental Figure S7) targeting the S segment [17]. Additional patients (*n* = 791) not initially subjected to NGS were screened by RT–PCR. Three more strong OROV PCR positives from Leticia (LET-352, LET-882, LET-814) were identified, for which full genome sequences were later obtained from LET-352 and LET-882. Patient serum was used to infect Vero cells and induced CPE in culture, from which OROV was successfully isolated (Figure 1(d)). Culture supernatant was then serially diluted and assessed by plaque assay to quantify the virus stock titres (Supplemental Figure S8). Having positively identified multiple Oropouche fever infections in Colombia, we endeavoured to understand the source and extent of its spread.

### Cryptic circulation and separate introductions of oropouche virus in Colombia

**Figure 2.**
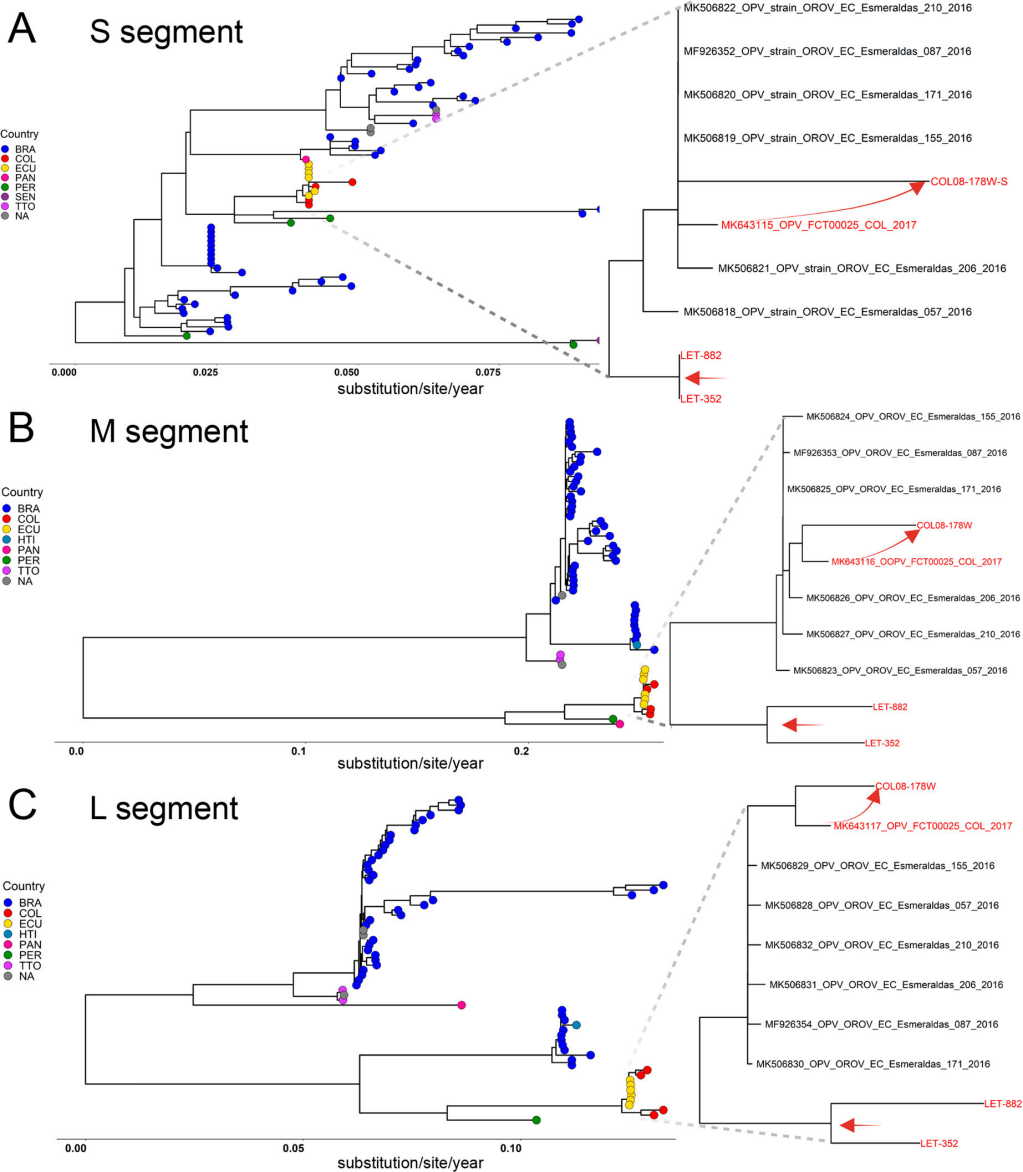
Independent introductions and cryptic circulation of Oropouche in Colombia cluster with Ecuadorian strains and all descend from Peru. ML phylogenetic trees of were reconstructed with three strains sequenced here (red) and all available Oropouche references in GenBank. Genetic distances are measured in substitutions/site/year for the A. S segment, B. M segment, and C. L segment. All nodes showed support value indicated by bootstrap >55. In all cases the tips were coded to represent the geographic location denoted in the legend. Curved-red arrows highlight the local evolutionary link between the initial case (FCT00025) reported in Turbaco and the 0200178W index from Cucuta. The straight red arrows represent the segregation of strains from Leticia which occurred before 2017 as an independent introduction directly from Peru.

All available OROV full genome sequences from South and Central America were retrieved from GenBank and aligned with 0200178W, LET-352, and LET-882 to reconstruct phylogenetic trees (Figure 2, Table S2). Each of the genome segments clustered with sequences reported in a 2016 outbreak in Esmeraldas from neighbouring Ecuador [11]. Within this clade, 0200178W from Cucuta branched closest to FCT00025, reported in 2017 from nearby Turbaco, Colombia [12]. The estimated genetic distances were 0.4%, 0.5% and 0.4% for the L, M and S segments, respectively. Longer branch lengths of 0200178W denote greater genetic diversification, to reveal that OROV has been cryptically circulating in Colombia and evolving locally (Figure 2(A–C)). By contrast, LET-352 and LET-882 from Leticia in the Amazon River Basin form a separate branch within this cluster absent other reported strains to suggest an independent introduction. With Peruvian strains ancestral to all these new sequences, it appears OROV arrived in the north of Colombia by way of Ecuador and to the south directly from Peru.

### Active and resolving OROV infections are detected in AFI specimens

**Figure 3.**
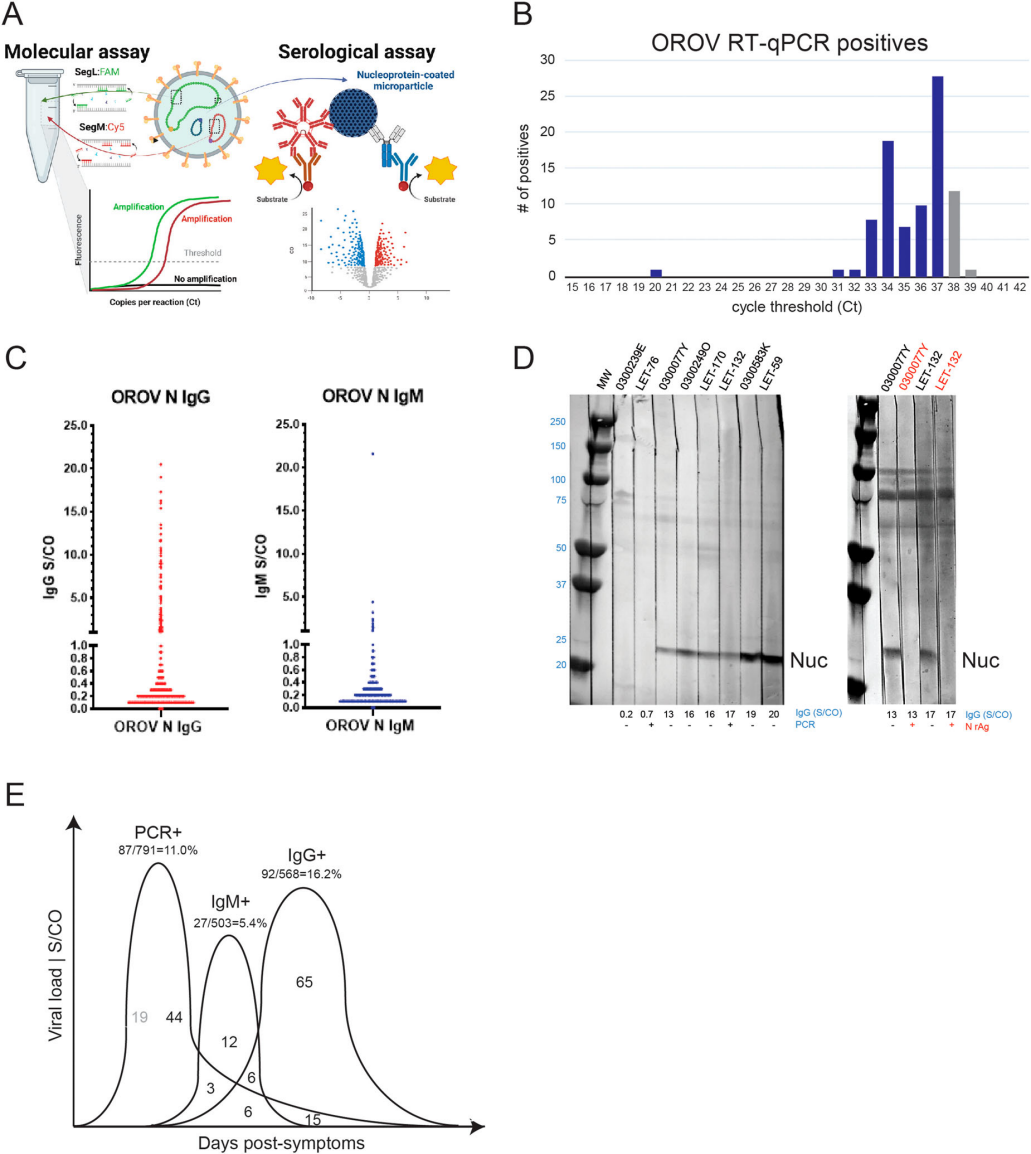
Numerous cases of Oropouche fever detected in Colombia. A. Genomic depiction of where primers/probes are situated on L and M segments for RT-qPCR. The nucleocapsid protein encoded on the S segment was used for serology. B. RT-qPCR results, plotting the number of viremic OROV cases versus cycle threshold (Ct). C. Anti-OROV nucleocapsid IgG (left) and IgM (right) antibodies detected Colombians with AFI. Greyzone (0.8-1.0) and S/CO ≥ 1.0 values were considered positive. D. (*left*) Western blot detection of OROV-infected Vero lysate using patient plasma (IgG S/CO values are indicated). The band at 25 kDa is nucleocapsid. (*right*) Competitive inhibition assay showing loss of anti-nucleocapsid reactivity in presence of excess recombinant protein (lanes 2, 4). E. Summary depiction of RT–PCR and antibody positives.

To determine the extent to which other febrile cases in Colombia were due to OROV, we developed an RUO RT-qPCR assay with dual detection of L and M segments (Figure 3(A), Supplemental Figures S1–S3). The linear range spanned 10^1^–10^9^ copies/ml with R^2^ values approaching 1.0 and efficiencies of ∼100% for both segments (Figure S4). Analytical sensitivity measured in water and plasma yielded a limit of detection of ∼ 2.5 copies/reaction and 0.6 PFU/ml, respectively, with Ct 38.3 ± 0.8 as a “grey-zone for the assay” (Figure S4, Table S5). In parallel, we developed an automated, RUO serological assay on the ARCHITECT for detection of anti-nucleocapsid antibodies and determined specificity on a population of 500 presumed OROV antibody-negatives from healthy, US donors (Figure 3(A), Figure S6).

A total of 791 febrile illness cases were screened by the Oropouche RT-qPCR assay. In an initial round of testing, we detected 68 positives, and following a freeze/thaw of the nucleic acid, we detected 28. Five patients were positive in both screens and 13 had signals in both channels. The overall OROV incidence was 10.9% (87/791). The majority had Cts ≥30 (corresponding to ≤ 10 PFU/ml), and many were only positive in the FAM (L) channel, consistent with the delay seen for M in the CY5 channel (Figure 3(B), Supp Table S6). Since these values are near the assay limit of detection, it is expected that the same sample could have conflicting results in both testing rounds. Grey zone samples were included in this total, while those having Ct ≥ 39 were excluded as false positives [31]. Overall, we believe the distribution of Ct values is an indication of having detected the tail end of viremia as infections are resolving. Notably, there were 10 cases of OROV and DENV co-infections in Leticia detected by RT-qPCR.

Anti-nucleocapsid reactivity was measured on the ARCHITECT for specimens with remaining volume: *n* = 568 for IgG and *n* = 503 for IgM. In sharp contrast to the US population, we detected 92 IgG (16.2%) and 27 IgM (5.4%) positives among the Colombian patients with AFI, of which 12 were dual IgM+/IgG+ (Figure 3(C), Supp Table S7). To verify measurements reflected anti-Oropouche responses, Western Blots were performed with patient plasma (1:100 dilution) reacted to OROV-infected Vero cell lysate resolved on SDS-PAGE. As expected, antibody-negative samples (S/CO < 1.0) had no reactivity, whereas IgG and IgM positives all detected a band at 25 kDa. The intensity of nucleocapsid reactivity tracked dose-dependently with S/CO values (Figure 3(D)- *left*). As a final demonstration of specificity, excess recombinant protein was incubated with two different patient sera prior to blotting. The addition of free nucleocapsid competed for binding to patient antibodies and inhibited detection of the 25 kDa band for both individuals (Figure 3(D)- *right*).

It is well established that OROV antibody levels are detectable within days of symptom onset, and thus we observed a strong overlap between viremia and humoral responses [32]. From the 87 PCR+, 68 of these were tested by serology, and 24 (35.3%) were antibody positive (Figure 3(E), Supp Table S7). As expected, the percentage with detectable viremia that were IgM+ was higher than IgG+: Nearly one-third (9/27; 33.3%) of IgM+ were PCR+ compared with one-fifth (21/92; 22.8%) of IgG+ being PCR+. There were 12 dual IgM+/IgG+ of which 50% (6/12) were PCR+. The majority (65/92; 70.6%) of IgG+ were PCR negative. We estimate the incidence of new infections range from 3-11% depending on coincident detection of antibodies. Similar estimates of 4.5%–20% are reached for recently resolved OROV infections. In summary, molecular and serologic testing indicates OROV infection was a significant contributor to febrile illness during the 2019–2021 timeframe.

### OROV epidemiology in Colombia

**Figure 4.**
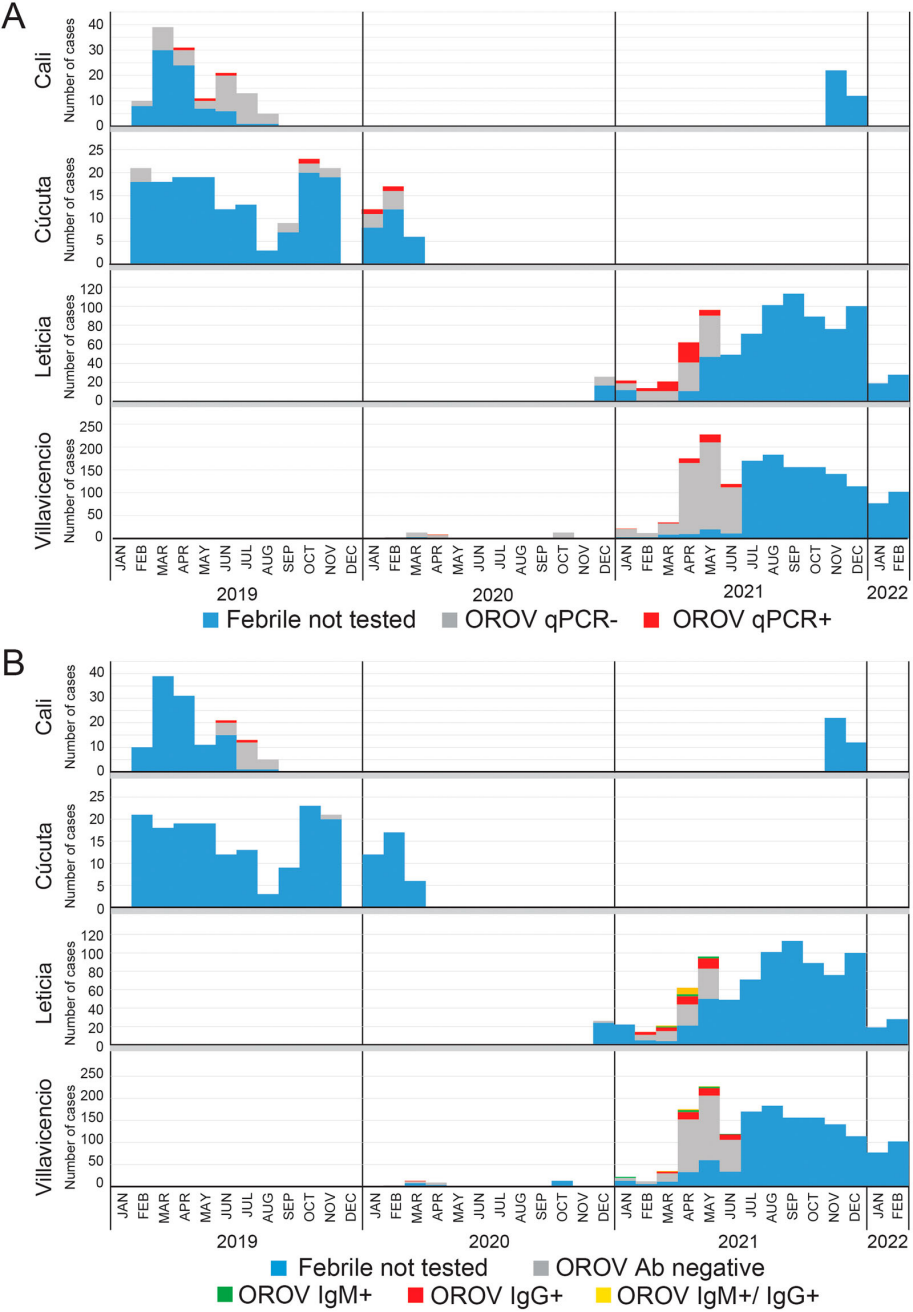
OROV cases are present throughout Colombia but concentrated near the Amazon River basin. A. OROV molecular results by year and month for each site. Stacked histograms indicate the number of RT-qPCR positives (red), RT-qPCR negatives (grey), and remaining AFI samples not tested (blue). B. OROV serology results by year and month for each site. Stacked histograms indicate the number of IgM positives (green), IgG positives (red), dual IgM/IgG positives (yellow), antibody negatives (grey), and remaining AFI samples not tested (blue).

To identify epidemiologic trends, we documented the locations and timeframes when active (e.g. RT-qPCR+) and resolving/resolved (e.g. IgM/IgG+) OROV cases were detected (Figure 4(A,B)). Out of 130 febrile cases from Cali during February-August 2019, 53 individuals were tested for OROV and only 3 (5.6%) RT-qPCR positives were detected: 1 each in May, June, and July. However, 22 samples tested by serology only included those from June-August, in which 2 (9.1%) IgG positive cases were found in June and July. While AFI cases (*n* = 193) in Cucuta spanned from February 2019-March 2020, OROV molecular testing in Cucuta was primarily restricted to samples collected between September 2019-February 2020 and three active cases were found in October, January, and February (3/19; 15.8%). Due to insufficient volume, only one Cucuta case was tested by serology and it was negative. In Villavicencio, there were relatively few (*n* = 38) febrile cases collected in 2020 for which we detected only 1 RT-qPCR positive out of 32 tested and 1 IgG+ out of 12 tested. The majority of Villavicencio samples (n =  1,510) were obtained in 2021 and 433 were screened by serology OROV assays while 534 samples were screened by RT-qPCR OROV assay. Viremic cases (*n* = 37/534; 6.9%) were consistently 5-7% per month over this interval. Screening by serology over the same time frame yielded similar month to month values, with overall percentages at approximately 14.8% (64/433). While most were IgG+ (*n* = 50), there were 10 IgM+ and 4 dual IgM+/IgG+. Finally, from the 887 AFI cases in Leticia, we screened 153 by OROV qPCR. As with Villavicencio, the testing occurred during the 1st half of 2021, but there were clearly more qPCR positive cases in March and April as compared to the three prior months: 31/71 (43%) – vs – 6/33 (18%). From 113 screened for antibodies, there were 35 IgG+ (31%) and 13 IgM+ (11.5%), 8 of which were dual positives. Therefore, there were more active (43/153; 28% by RT–PCR) and seroconverted (48/113; 42.4%) OROV infections in Leticia compared to the other sites (Table 1). The rainy season spans April to May and October to November, and the dry season usually occurs in December to January and July to August. While OROV testing was not applied there evenly throughout the year, the spike in cases observed coincided with the rainy season (March-May).

**Table 1.** Epidemiologic trends of Dengue and Oropouche infections in Colombia. Infections for each virus were categorized by age, sex, location, and climate phase. Clinical manifestations associated with each infection are listed. *P-*values (<0.05) denote statistical significance between cases and non-cases among the categories.

Characteristic	Category	Dengue				Oropouche			
		Positive (n)	Total tested (n)	% Cases	*p-*value	Positive (n)	Total tested (n)	% Cases	*p-*value
Age (years)	<18	140	288	48.6	<0.01	9	64	14.1	0.18
	18–29	210	1160	18.1		32	299	10.7	
	30–39	125	692	18.1		21	189	11.1	
	40–49	73	402	18.2		18	109	16.5	
	50–59	39	269	14.5		18	90	20.0	
	>60	28	156	17.9		7	41	17.1	
Sex	Male	337	1651	20.4	0.65	55	401	13.7	0.77
	Female	278	1316	21.1		50	391	12.8	
Location	Cali	17	164	10.4	<0.01	3	53	5.7	<0.01
	Cúcuta	112	193	58.0		3	19	15.8	
	Leticia	218	887	24.6		48	153	31.4	
	Villavicencio	268	1723	15.6		51	567	9.0	
Year of sample collection	2019	104	288	36.1	<0.01	4	63	6.3	0.05
	2020	49	99	49.5		3	51	5.9	
	2021	417	2358	17.7		98	678	14.5	
	2022	45	222	20.3		0	0	0	
Climate phase	El Niño	84	201	41.8	<0.01	3	31	9.7	<0.01
	La Niña	378	1887	20.0		62	334	18.6	
	Neutral	153	879	17.4		40	427	9.4	
Headache	Yes	573	2819	20.3	0.02	94	748	12.6	0.03
	Not reported	42	148	28.4		11	44	25.0	
Muscle pain	Yes	532	2702	19.7	<0.01	96	740	13.0	0.49
	Not reported	83	265	31.3		9	52	17.3	
Odynophagia	Yes	139	772	18.0	0.03	29	126	23.0	<0.01
	Not reported	476	2195	21.7		76	666	11.4	
Retroorbital pain	Yes	399	2025	19.7	0.04	69	569	12.1	0.16
	Not reported	216	942	22.9		36	223	16.1	
Red eyes	Yes	136	602	22.6	0.22	28	148	18.9	0.03
	Not reported	479	2365	20.3		77	644	12.0	
Chills	Yes	497	2444	20.3	0.28	83	666	12.5	0.16
	Not reported	118	523	22.6		22	126	17.5	
Abdominal pain	Yes	325	1364	23.8	<0.01	33	252	13.1	1
	Not reported	290	1603	18.1		72	540	13.3	
Weakness	Yes	372	1650	22.5	<0.01	76	534	14.2	0.29
	Not reported	243	1317	18.5		29	258	11.2	
Skin Rash	Yes	139	333	41.7	<0.01	13	116	11.2	0.57
	Not reported	476	2634	18.1		92	676	13.6	
Dizziness	Yes	303	1524	19.9	0.24	34	513	6.6	0.58
	Not reported	294	1475	19.9		71	279	25.4	
Vomit	Yes	321	1492	21.5	0.29	44	419	10.5	0.02
	Not reported	297	1491	19.9		61	373	16.4	
Petechiae	Yes	19	25	76.0	0.25	0	3	0.0	0.95
	Not reported	596	2942	20.3		105	789	13.3	
Diarrhoea	Yes	134	596	22.5	<0.01	16	126	12.7	1
	Not reported	481	2371	20.3		89	666	13.4	

An assessment of other OROV epidemiologic trends was made in comparison to dengue (Table 1). When comparing cases to non-cases or symptoms reported vs non-reported, dengue status by age, location, year, climate phase, and eight symptoms (headache, muscle pain, odynophagia, retro orbital pain, abdominal pain, weakness, skin rash, and diarrhoea) was statistically different (*p* < 0.05), while Oropouche status by location, year, climate phase, and six symptoms (headache, odynophagia, retro orbital pain, red eyes, chills, and vomit) were statistically different (*p* < 0.05). After logistic regression analysis, weakness (OR = 1.3; CI95% = 1.1–1.6), skin rash (OR = 2.3; CI95% = 1.8–3.1) and petechiae (OR = 3.6; CI95% = 1.4–9.5) were risk factors associated with dengue cases (Supp Table S9), while the location (OR = 2.6; CI95% = 1.8–3.8), climate phase (OR = 1.7; CI95% = 1.1–2.5) and odynophagia (OR = 2.0; CI95% = 1.2–3.4) were risk factors associated with Oropouche cases (Supp Table S10). Multicollinearity nor plausible interactions were not identified. Logistic regression models were rebuilt excluding climate phase, collection site, and time of collection to focus solely on clinical symptoms and demographic factors. Variables positively correlated with increased odds for dengue (Supp Table S11) were people aged <18 yr (OR 3.24; CI95% 2.0–5.2), red eyes (OR 1.6; CI95% 1.2–2.1), weakness (OR 1.21; CI95% 1.1–1.5), skin rash (OR 2.2; CI95% 1.7–2.9), and petechiae (OR 3.5; CI95% 1.3–9.2). Variables positively correlated with increased odds of Oropouche (Supp Table S12) were odynophagia (OR 3.2; CI95% 2.4–4.3), abdominal pain (OR 2.5; CI95% 2.0–3.0), and sex-female (OR 1.3; CI95%1.1–1.6). The results of each analysis were generally in agreement, wherein, we observed trends, symptoms, and potential risk factors that distinguished arboviruses sharing overlapping clinical presentations.

## Discussion

Colombia has been identified as an emerging infectious disease hotspot [33]. The country is periodically afflicted by epidemic waves of vector-borne diseases such as dengue, yellow fever, chikungunya, and Zika viruses, among others. We conducted a facility-based fever monitoring study at designated clinics/hospitals at four sites in Colombia (Cucuta, Cali, Villavicencio, Leticia) with the primary objective of observing the clinical and laboratory characteristics of AFI without an identified infectious cause. From a subset of AFIs sequenced by NGS, we identified an active case of Oropouche virus in Cucuta highly related to a strain previously isolated in 2017 [12]. Conventional PCR identified three additional OROV cases in Leticia. To understand the magnitude and geographic distribution of OROV infections in Colombia, we proceeded to develop RT-qPCR and serologic assays to screen the AFI cohort. Our collective results suggest OROV is widely circulating in Colombia and is a significant cause of undifferentiated acute febrile illness.

A greater understanding about the spread of OROV in Colombia was deduced from our phylogenetic analyses. To begin, the high identity of 02000178W to FCT00025 from Turbaco along with its comparatively longer branch length indicates OROV has been circulating in northern Colombia now for several years [12]. The clustering of these sequences with Esmeraldas strains further suggests they originated from the Ecuador outbreak in 2016. Next, the two full genomes we characterized from Leticia in the south were also from lineage 2 and clustered with the Ecuador strains, however, they formed a unique branch indicative of an independent introduction. With Peruvian strains basal to all of these, it suggests a common source. Finally, genetic reassortment among orthobunyaviruses of the same serogroup occurs frequently in nature and has led to the emergence of new viruses, occasionally with increased virulence. Indeed, OROV reassortants have been reported in neighbouring Peru such as Iquitos and Madre de Dios viruses [34]. The consistent branching patterns for each of the genome segments sequenced in our study relative to each other and Ecuadorian strains indicates reassortment was not a factor.

Finding these initial OROV strains prompted the development of a RT-qPCR assay to facilitate screening of additional AFI samples. A dual-target detection strategy was deployed to accommodate high genetic diversity in the Americas, including detection of reassortants, and ensure assay performance [35,36]. While recent RT-qPCR assays have been developed targeting the S gene [31,37], our phylogenetic analysis determined that the S segment contains less phylogenetic structure, is more susceptible to polymorphisms, and exhibits a lack of congruence with M and L segments. This assessment concurs with the recent report by Gutierrez et al. [35] which found a high level of uncertainty in S phylogeny that suggests a different evolutionary rate for this segment compared to M and L [35]. Our serology assay strategy allowed us to detect resolving (IgM) and resolved (IgG) OROV infections. Nucleocapsid proved easy to express and purify from bacteria and was readily adapted to high-throughput screening on the ARCHITECT [30]. Based on western blots of cell culture lysates with patient sera, this protein is also highly abundant and immunogenic. With these approaches used in conjunction, we observed an overall viremia incidence of 10.9% and an antibody prevalence of 5.4% for IgM and 16.2% for IgG. These values varied according to geography and time of year, with Leticia having the highest rates, but the implications are clear that Oropouche is indeed circulating in Colombia despite few to no previous reports.

During acute OROV disease, viremia peaks on day 2 post-symptom onset and decreases rapidly, in which case viral RNA may be cleared by the time a patient seeks care [38]. In roughly 60% of clinical cases after the fever subsides, disease is known to recur. Given that similar symptoms are manifested, OROV is often misdiagnosed and consequently both scenarios will lead to under-reporting [39,40]. The analytical sensitivity of 2.5 copies for our RT-qPCR assay (95% confidence) was a critical factor in the detection of active cases. Taking into consideration the window for the development of clinical symptoms and the viremic phase of OROV are both relatively short [41], together with the assumption that in endemic regions some individuals might have partial immunity thereby decreasing their viral loads, then less sensitive assays could be prone to false negative results. Indeed, Rojas et al. [37] assessed 100 clinical samples from patients with acute febrile illness following an average of 3.5 days after symptom onset, with all samples testing negative. Our sensitivity was essential since most positives we encountered had viral loads of ≤10 copies per reaction (Figure 3(B)). Importantly, these weakly positive molecular results were bolstered by serology data which found that 35.3% of these individuals had anti-OROV antibodies (Figure 3(E)). We note here the frequent detection of RNA among IgG reactive suggesting prolonged shedding can occur following seroconversion. This is consistent with findings that IgM and IgG antibodies are generated 1 day to 2 weeks after disease onset [42]. With an automated antibody assay, large serosurveys are now possible to gain a clearer picture of the prevalence throughout the Americas and in animal reservoirs.

In our study, we detected of few cases (*n* = 10) of OROV-DENV co-infections in Leticia. As this region in the Amazon basin is hyperendemic for dengue and endemic for other arboviruses, there is a need to study the outcomes of potential dual infections. A previous study reported a frequency of at least 7% of co-infections and they occurred more frequently during the dry season [43]. In tropical countries like Colombia, the co-circulation of arboviruses can increase the frequency of coinfections in humans. This scenario raises the likelihood that vectors could be also exposed to multiple pathogens during one feeding episode. The impact of coinfection on vector species has been studied, showing that vectors can be co-infected and transmit all combinations of arboviruses simultaneously [44], increasing the risk of simultaneous spread of pathogens. Arbovirus coinfection and co-transmission in tropical areas could be silently growing as a neglected public health problem in Latin America.

Several fever surveillance studies conducted in Latin American countries have indicated that a high proportion of febrile cases remain undiagnosed. Here in our study, combining DENV and OROV cases, we are still left with more than half of AFI cases going undiagnosed. Patients with undifferentiated AFI may also have common and atypical infections for which testing is not normally available. Thus, despite the known limitations, increased surveillance and implementation of novel laboratory detection methods such as metagenomic sequencing is highly advised. Oropouche and other arboviruses are on the rise in Brazil and throughout South America [3,45]. While previous work has reported the presence of OROV in Colombia [12], our study demonstrates active circulation in the four field sites and suggests that this virus is causing more infections than previously reported in other regions of the country. Indeed, our OROV serology prevalence explains why many cases are negative by molecular methods. Accurate detection of virus activity in vectors, humans, and animal populations are crucial components of effective control, prevention, and management of vector-borne diseases. The methods used in our work for viral and serological detection of OROV should be scaled up commercially and implemented as routine standard of care when evaluating patients with AFI and contribute to a better understanding of the disease burden of OROV in the region.

## Supplementary Material

Supplemental Material

## Data Availability

The authors confirm that the data supporting the findings of this study are available within the article and its supplementary materials. Raw data and other additional information are available from the corresponding author upon request.
