# Supplementary material for: Oropouche virus as an emerging cause of acute febrile illness in Colombia
Source: Emerg Microbes Infect. 2022 Oct 14;11(1):2645–57. doi: 10.1080/22221751.2022.2136536 (PMC9639516; doi:10.1080/22221751.2022.2136536)
Supplement: Supplemental Material [file TEMI_A_2136536_SM5082.docx]

Online Supplemental Information

Oropouche virus as an Emerging Cause of Acute Febrile Illness in Colombia

Karl A Ciuoderis^1,4*^, Michael G. Berg^2,4*^, Lester J. Perez^2,4^, Abbas Hadji^2,4^, Laura S. Perez^1,4^, Leidi Carvajal^1,4^; Kenn Forberg^2,4^, Julie Yamaguchi^2,4^, Andres Cordona^1,4^, Sonja Weiss^2,4^, Xiaoxing Qui^2,4^, Juan Pablo Hernandez^1,4^, Francisco Averhoff^2,4^, Gavin A. Cloherty^2,4^, Jorge E Osorio^1,3,4^

*co-first author

^1^Colombia/Wisconsin One Health Consortium (CWOHC), Universidad Nacional de Colombia, Medellín, Colombia.

^2^Infectious Diseases Research, Abbott Diagnostics, Abbott Park IL USA

^3^Department of Pathobiological Sciences, School of Veterinary Medicine, University of Wisconsin, Madison, WI

^4^Abbott Pandemic Defense Coalition

Table of Contents

Materials and Methods…………………………………………………..……… 2-6

Figures 1-5 RT-qPCR assay development……………………………………….6-10

Figures 6-8 Serology, RT-PCR, virus culturing………………………………...11-13

Table 1 RT-PCR primers and literature references…………………………………14

Table 2 GenBank accessions for phylogenetics…………………………………15-17

Tables 3 & 4 RT-qPCR assay conditions…………………………………….……..18

Table 5 RT-qPCR dropout with virus lysates……………………………….………19

Table 6 List of RT-qPCR positives……………………………………….……..20-22

Table 7 List of serology positives………………………………………….………..23

Tables 8-12 Logistic regression analysis…………………………………….…..24-25

Online Supplemental Materials and Methods

## Study area and population.

All study sites had a warm and humid tropical climate with an average annual temperature of 26.4°C (IQR= 2.37; median= 26.4) and an average total annual rainfall of 2,451 mm (IQR= 2,314.9; median= 2,399.1). Average mean altitude of 475.3 meters above the sea level (IQR= 340.8; median= 393.5). Climate variations are characterized by rain and temperature fluctuations. The annual rainfall in Colombia varies widely based on altitude. May to October registers the highest amount of precipitation, however, this pattern varies considerably between regions. The western Andes and southeastern Colombia receive abundant rainfall throughout the year. Colombia is strongly influenced by El Niño/Southern Oscillation (ENSO) climate phenomena with two phases of a recurring climate pattern: El Niño (warmer and drier weather) and La Niña (colder and rainy weather) (Munoz et al. 2021).

## Multiplex Zika, chikungunya, and dengue (ZCD) real time RT-PCR

Viral RNA was extracted from 150 µl of serum using Zymo Quick-RNA Viral Kit (Zymo research, USA) following manufacturer's instructions. RNA was eluted in 30 µl nuclease-free water. RNase P (RP) gene was used as an endogenous control. Primers and probes used are shown in **Supplemental** **Table S1**. SuperScript™ III One-Step RT-PCR System with Platinum™ Taq DNA Polymerase was used for amplification. Cycling conditions were as follows: 50°C for 30 min, 95°C for 2 min and 45 cycles of 95°C for 15 sec and 60°C for 1 min. The positive results were those in which a positive amplification curve was observed before cycle 40.

## NGS Sequencing

Following pre-treatment of serum with benzonase, total nucleic acid extractions were performed on the Abbott m2000sp instrument. A positive control library containing a cocktail of viruses (Parechovirus A, AdV-4, Rotavirus A, VZV, AAV-2) spiked at log 4.0 cp/ml into normal/uninfected human plasma and a negative control lacking these were prepared in parallel. All manipulations were performed in 96 well plates using an epMotion liquid handler (Eppendorf). Reverse transcription with SSIV 1st Strand reagents (Life Technologies, Carlsbad, CA, USA) for cDNA synthesis was primed with random hexamers and oligo(dT), after which the 2^nd^ strand was copied with Sequenase V2.0 T7 DNA pol (Affymetrix, Santa Clara, CA, USA). Following bead clean up, double-stranded cDNA was ‘tagmented’ with Nextera XT (Illumina, CA, USA). Libraries were quantified on a Qubit (Invitrogen, USA) and BioAnalyzer Tapestation 2200 (Agilent, USA).

## Conventional OROV RT-PCR

Viral RNA was extracted from 150 µl of serum using Zymo Quick-RNA Viral Kit (Zymo research, USA) following manufacturer's instructions. RNA was eluted in 30 µl nuclease-free water. Primers and probes used are shown in **Table S1**. The iTaq Universal Probes One-Step Kit was used for amplification. Cycling conditions were as follows: 50°C for 10 min, 95°C for 3 min and 40 cycles of 95°C for 30 sec, 55°C for 60 sec and 72°C for 1 min. A final extension of 72°C for 10 minutes was used. Each PCR product (5 ul) was subjected to 1.5% agarose gel electrophoresis and the amplified DNA was visualized by UV light after staining with 1X SYBR safe DNA Gel Stain (Invitrogen, USA). The size of the amplified DNA fragments was determined by comparison with the molecular size marker Quick-Load Purple 50 bp DNA Ladder (New England BioLabs, USA). To monitor the quality of extraction and presence of PCR inhibitors, each sample was also tested for the amplification of the housekeeping Ribonuclease P (RP) gene (Fan et al. 2014). Thermocycling conditions were 50°C for 10 min, 95°C for 3 min, followed by 40 cycles of 95°C for 15 sec and 58°C for 30 sec.

## Cell culture isolation, plaque assay and virus titration

For virus isolation, C6/36 HT cells were cultured in L-15 medium with 5% FBS and Vero cells were cultured in DMEM medium with 5% FBS. When cells reached 85% confluence, serum samples were inoculated as a 1:80 dilution (serum: media). Cells were incubated at 34°C (C6/36 HT cells) or 37°C with 5% CO_2_ (Vero cells) for 1 hour (hr) after which the inoculum was replaced with fresh culture media and incubated for 7 days with daily observation. Plaque assays were performed using Vero cells (80,000 cells/well) in 24-well plates. Following a 72 hr incubation the medium was discarded, cells were washed with PBS, and 500 µl of 4% paraformaldehyde was added to fix the cells. After 2 hr, 0.5% crystal violet was then added for 1 hr to stain cells, washed with water, and virus titers were calculated.

## Development of new OROV molecular and serologic detection methods

We developed an RUO RT-qPCR Taqman assay with dual target detection for high-throughput screening on the *m*2000. Phylogenetic tree topologies for complete genomes L and M segments (**Figure S1**) and alignments (**Figure S2**) suggested ideal targets and were labeled with FAM and CY5, respectively (**Table S2**). Quantitative standards for both genomic segments were prepared by *in vitro* transcription. Targeted regions were cloned into the pBlueScript plasmid (**Figure S3**), linearized with *HindIII*, and RNA was generated with the MEGAscript kit (Ambion, Austin, TX). The size and quality of RNA were assessed using an RNA ScreenTape (Agilent, Santa Clara, United States) (**Figure S3**).

Serial dilutions of *in vitro* transcripts in water were detected by the respective primer/probe sets to determine linearity and efficiency (**Figure S4-***left*). The linear range spanned 10^1^-10^9^ copies/ml with R^2^ values approaching 1.0 and efficiencies of ~100% for both segments. Experiments were repeated with serial dilutions of virus lysates (C6/36 and Vero) spiked in plasma which yielded efficiencies of 112% for L and 102% for M (**Figure S4-***right*). Two-fold serial dilutions near the limit of detection established Ct 38.3±0.8 as a ‘grey-zone for the assay (**Supp Table S5**). Analytical sensitivity measured in water using 10 replicates from the three lower dilutions followed by a probit function estimation yielded a limit of detection of ~ 2.5 copies/reaction for each target region (**Figure S4-***left*). In plasma, the limit of detection was calculated as 0.6 PFU/ml (**Figure S4-***right*).

### ***RNA Secondary Structure***

RNA from the original plasma sample of index patient (0200178W) had a Ct=20.67 for L and a Ct=23.23 for M, for an estimated titer of log 4 cp/ml (**Supp** **Table S6**). Since the amplification for both target regions showed the same linear range and analytical sensitivity, we evaluated the role of RNA secondary structure to explain the shift in Ct for M compared to L. Indeed, the lower predicted free energy for M (-1075) compared to L (-1661) decreases RNA stability and likelihood of detection in clinical samples (**Figure S5).** The sequences from OPV/TRVL9760, OPV_OROV/EC/Esmeraldas057/2016 and OPV/BeH/543760 strains were used to obtain a consensus secondary structure for M (accession number: KC7591236, MK506823 and MG747576) and L (accession number: KC759122, MK506828 and MG747577) segments.

### ***Multiplex RT-qPCR, limit of Quantification, Efficiency, and analytical sensitivity***

Optimal reaction compositions were determined by the minimal concentration of primers and probes that guaranteed maximal amplification efficiency. Reactions were conducted in a final volume of 25 µL containing AgPath-ID™ One-Step RT-PCR reagents (Thermofisher Scientific, United States), 5 µL of RNA template, 0.4 µM target probe, 0.5 µM specific primers, and IC primer/probes concentrations following manufacturer recommendations (**Table S4**). A fast, two-step cycling thermal profile was used with an initial reverse transcription/denaturation/activation step of 45^ο^C for 10 min and 95^ο^C for 15 min, followed by 40 cycles of denaturation/annealing/elongation with acquisition of fluorescent data at 95^ο^C: 15 sec; 60^ο^C: 35 sec.

***Recombinant protein expression and purification***

Recombinant nucleoprotein was expressed in BL21 Star (DE3) chemically competent *E. coli* cells (Invitrogen) following isopropyl β-D-thiogalactopyranoside (IPTG) induction. E. coli cells were harvested by centrifugation (5,000 x g, 4 °C, 15 min) and the sediment was resuspended in cold extraction buffer (50 mM Tris/HCl pH 7.8, 200 mM NaCl, 10% Glycerol, 10mL imidazole). After addition of 0.1 mg/mL lysozyme, 1mL PMSF, 3U DNAse and incubation for 30 min the cell suspension was sonicated 3 times 15 seconds, followed by centrifugation twice (5,000 x g, 4 °C, 20 min). The supernatant was passed at 0.5 ml/min through a 1 ml Histrap HP column (Cytiva) preloaded with nickel ions and equilibrated in running buffer (50 mM Tris/HCl pH 7.8, 200 mM NaCl, 10% Glycerol). The bound protein was eluted at 1 ml/min with a 0–500 mM linear imidazole gradient. Fractions containing the purified protein as assessed by Coomassie Blue staining were pooled and dialyzed twice against 1 L of Tris-buffered saline (TBS, 50 mM Tris-HCl, 150 mM NaCl, pH7.8) over a 24 h period at 4°C.

***Prototype ARCHITECT ORO-N IgG or IgM assay***

We developed an automated, RUO serological assay on the ARCHITECT for detection of anti-nucleocapsid antibodies. The ARCHITECT Oropouche IgG or IgM cutoffs were set at S/CO=1.0: Serum/plasma specimens from a population of 500 presumed OROV antibody-negatives from healthy US donors (Gulf Coast Regional Blood Center) were screened to determine specificity and calculate the cut-off; samples ≥1.0 S/CO are considered reactive (**Figure S6**). The mean number of RLU and the standard deviation of the population was determined. A provisional cutoff was set at 13800 RLU for IgG and 4027 RLU for IgM, which is equivalent to the mean +7X the standard deviation of the negative population. There were 5 presumed ‘false positives’ for IgG (*left*) and 1 for IgM (*right*), for specificities of 99.0% and 99.8% (**Figure S6**). The same provisional cutoffs (S/CO of ≥1.0) were applied to the Colombian cohort.

***Western Blot analysis***

Cells were lysed with 10 mM Tris-HCl (pH 7.5) buffer containing 150 mM NaCl and 1% SDS at 100° C for 10 min. The viral lysate (80 μg/gel) was separated by electrophoresis on a 4-12% Criterion XT precast gel (BioRad) in the presence of sodium dodecylsulfate (SDS). The protein bands on the gel were electrophoretically transferred to a nitrocellulose membrane according to the manufacturer’s instructions (BioRad Trans-Blot Turbo Transfer Pack). After blocking with T20 blocking buffer (ThermoFisher), the nitrocellulose membrane was cut into 2 cm strips. WB strips were incubated with human plasma samples diluted in antibody buffer 1:200 overnight at 2-8°C. After removal of unbound antibodies, WB strips were incubated with Blotting Grade Affinity Purified Goat Anti-Human IgG (H+L) Alkaline Phosphatase Conjugate (BioRad) for 1 hour at room temperature. The strips were washed as described and Colorimetric AP substrate reagent kit solution was added (BioRad) (Coller et al. 2016).

***Climate data***

The El Niño Oceanic Index (ONI) and the Multivariate El Niño-Southern Oscillation (ENSO) Index (MEI) are the main variables for tracking seasonal climate patterns globally. These macroclimatic indexes were used to classify monthly climate conditions and evaluate the association of these phenomena with dengue cases. The MEI and ONI data were retrieved from the United States Office of National Oceanic and Atmospheric Administration (NOAA) Meteorological Service. According to the MEI and ONI data values, seasonal climate patterns or phases were classified as follows: El Niño (warmer/drier conditions), La Niña (colder/rainy conditions) and Neutral (means neither El Niño nor La Niña conditions were present in both the ocean and the atmosphere).

**
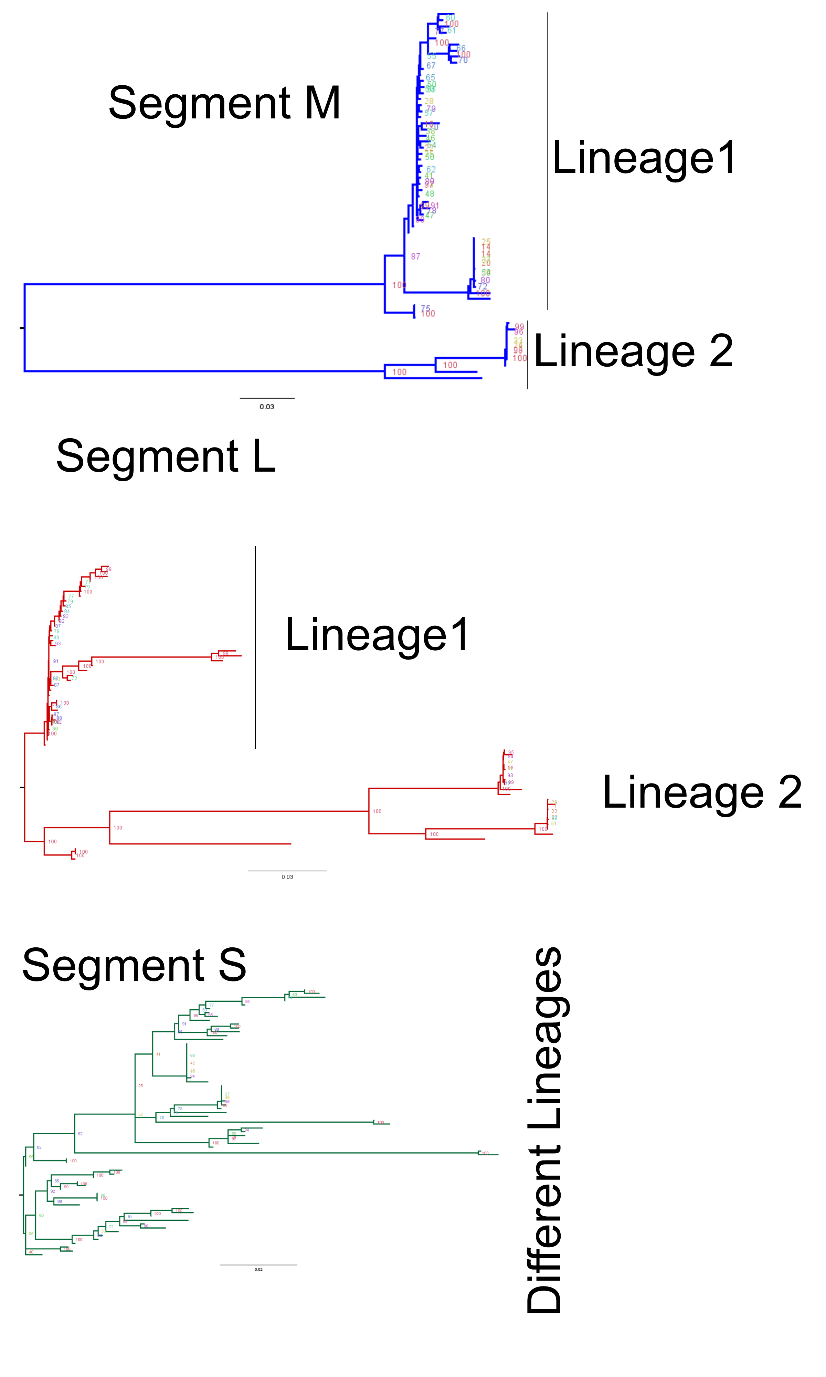
**

**Figure S1. Phylogenetic trees of Oropouche bunyavirus genome segments.** Maximum likelihood phylogenetic trees were reconstructed for each segment using all non-redundant genomes available in GenBank. The main lineages for L and M segments are labeled, while the lack of lineage demarcation observed for Segment S is also denoted.


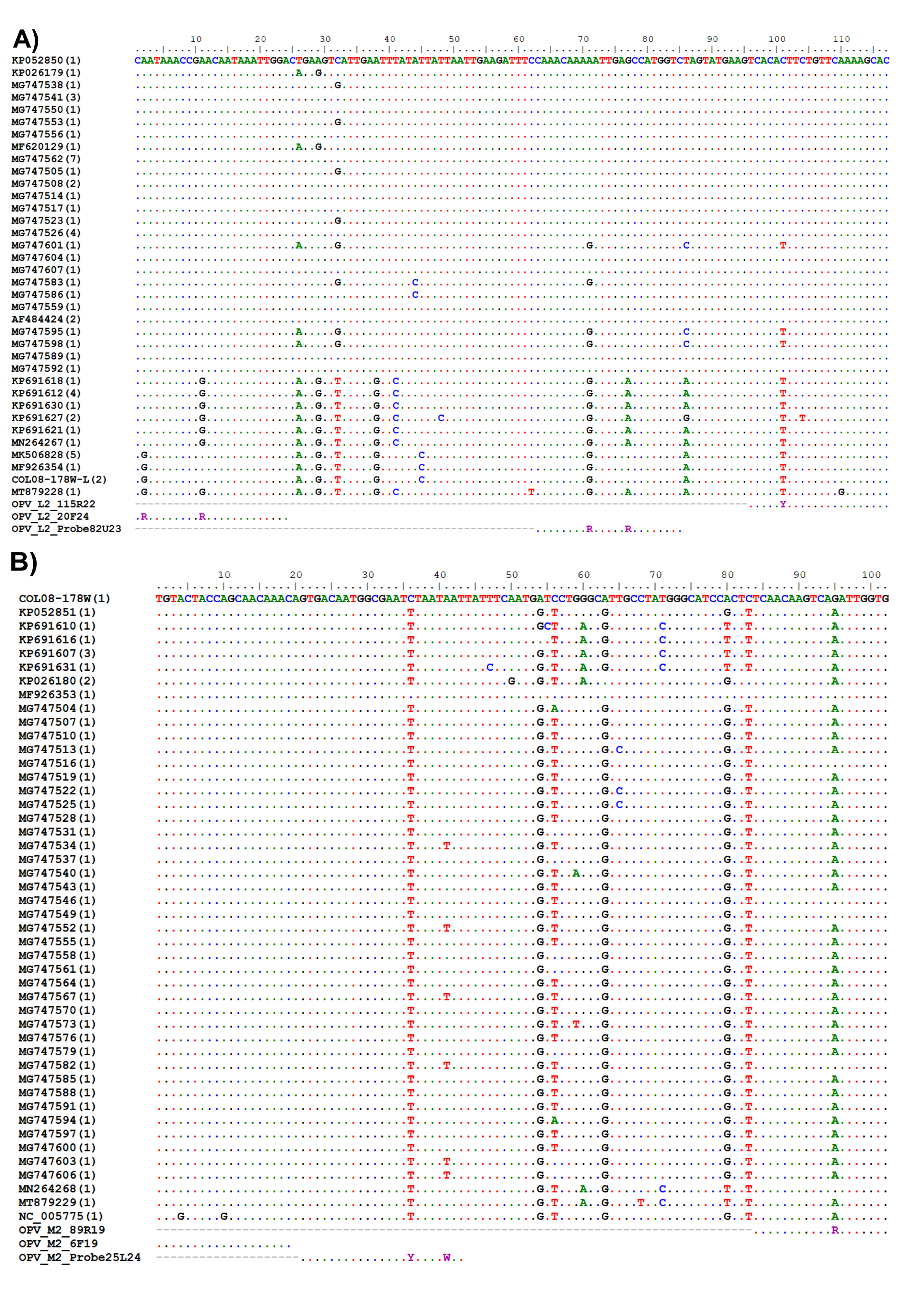


**Figure S2. *In silico* analysis for target region selection.** Alignments of target regions are shown for A) L segment and B) M segment of Oropouche bunyavirus. Sequences of primers and probes are shown beneath unique strains. All unique polymorphisms were included in degenerate primer/probe sequences and incorporated into *in vitro* transcripts for evaluation.


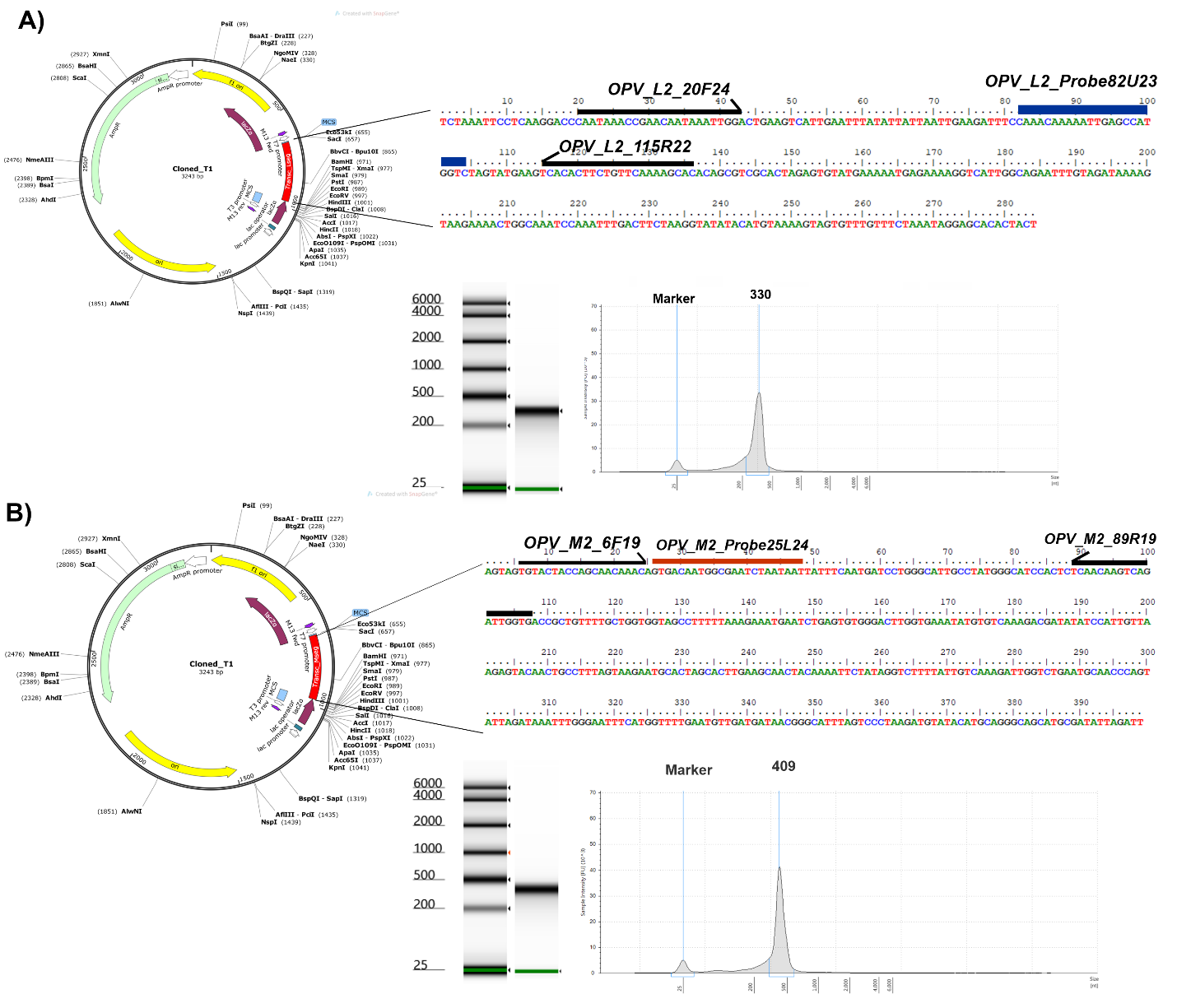


**Figure S3. In vitro transcripts for both segments of Oropouche bunyavirus.** pBlueScript plasmid constructs*,* the flanking target region, and capillary electrophoresis for each transcript is shown for the A) L segment and B) M segment. In the plasmid map, the sequences of primers and probes within the target region are denoted. Transcript sizes obtained after digestion are shown by gel ladder and electropherogram.

**
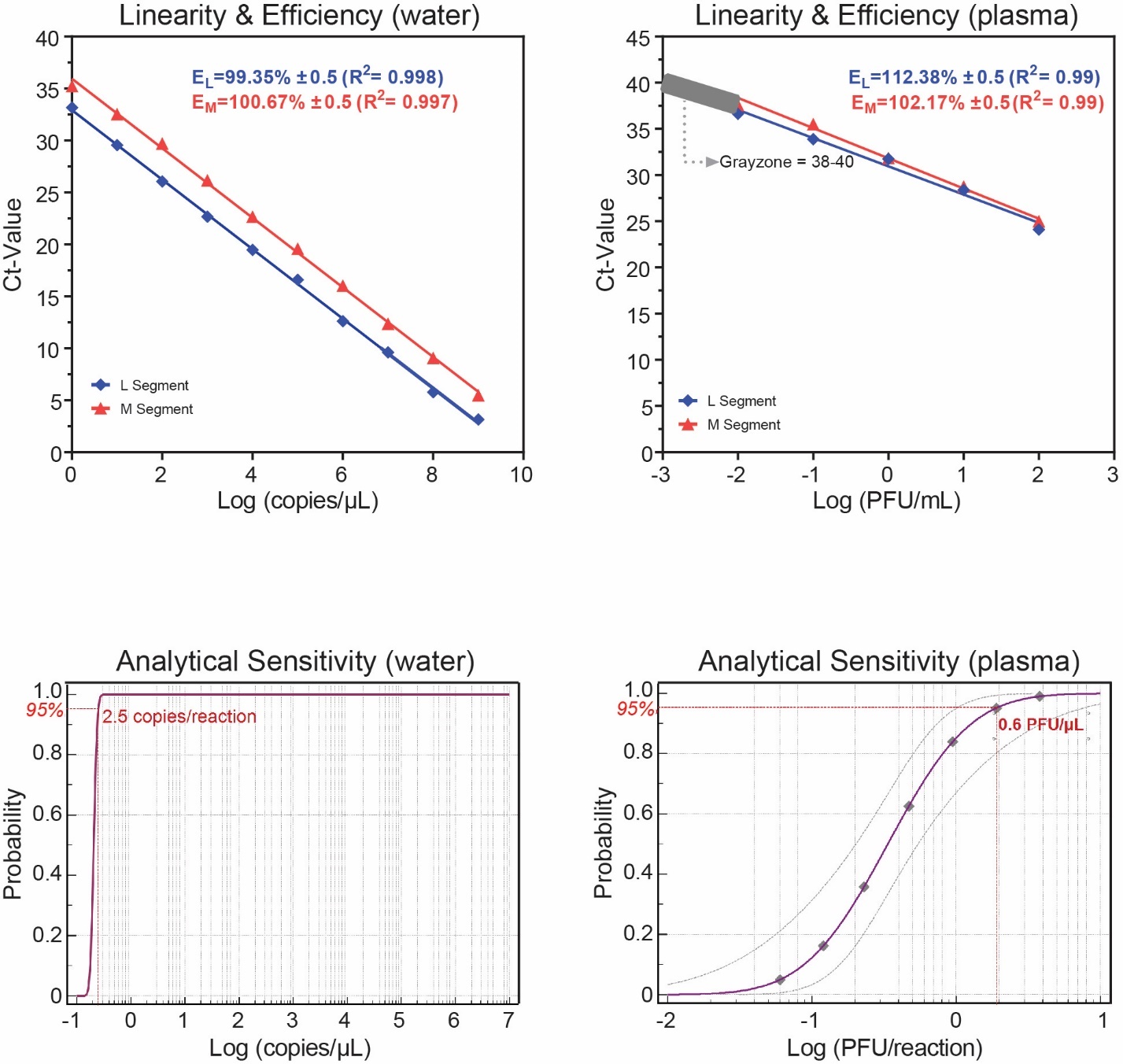
**

**Figure S4. Development of molecular diagnostics for Oropouche.**  **Top** Amplification curves evaluation showing the linear range of the assays for RdRp (L; blue) detected in FAM channel (*left*) and Gn (M; red) detected in CY5 channel (*right*). Serial dilutions of transcripts in water (*left*) and virus lysate in patient plasma (*right*) are plotted with calculated copy number and PFU/ml units versus Ct values. Ct’s above 38 in patient samples were considered greyzone **Bottom** Analytical Sensitivity detected with 95% of confidence for RdRp (L) and Gn (M) with transcripts in water (*left*) and virus lysate in patient plasma (*right*).


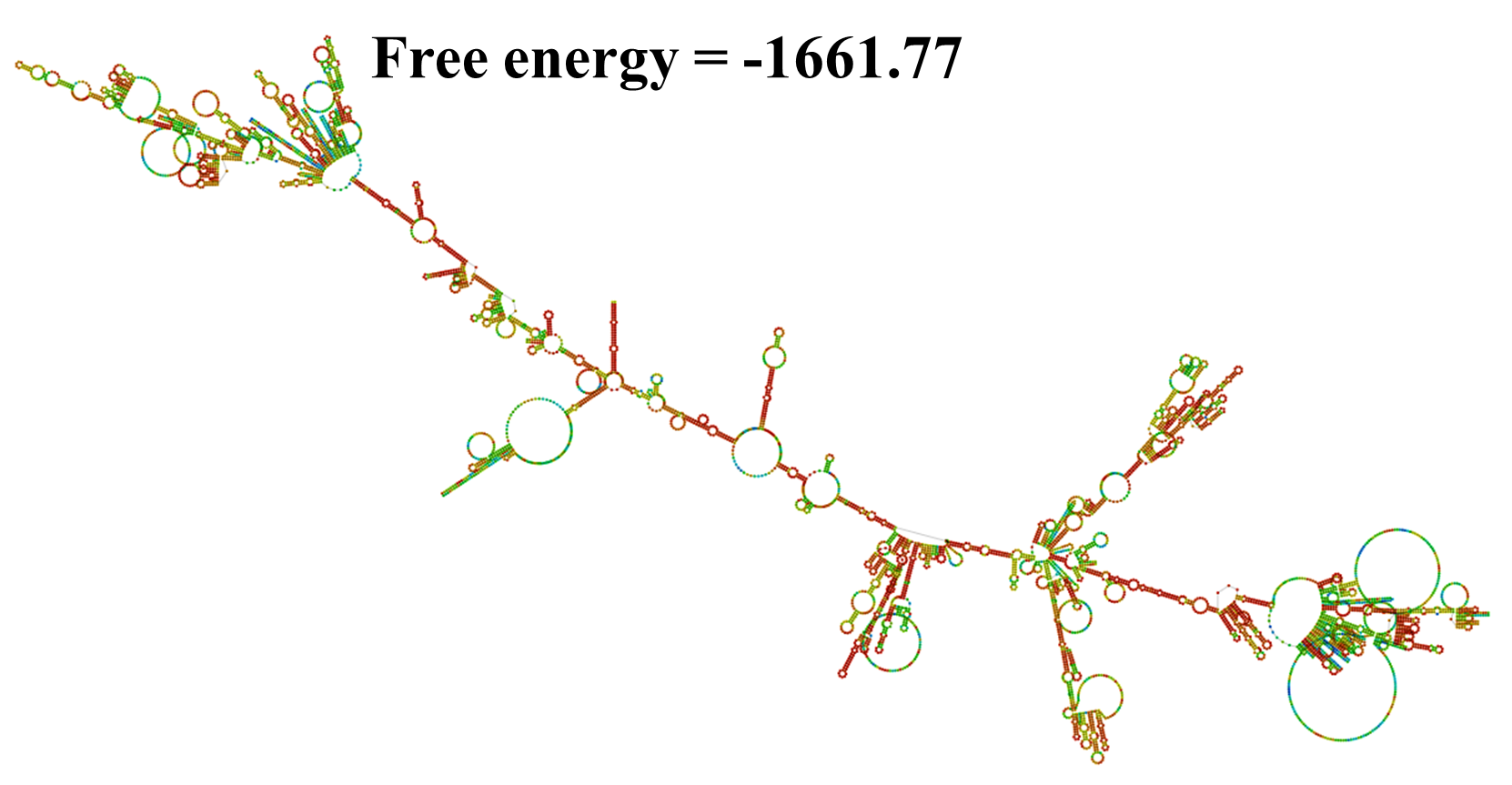

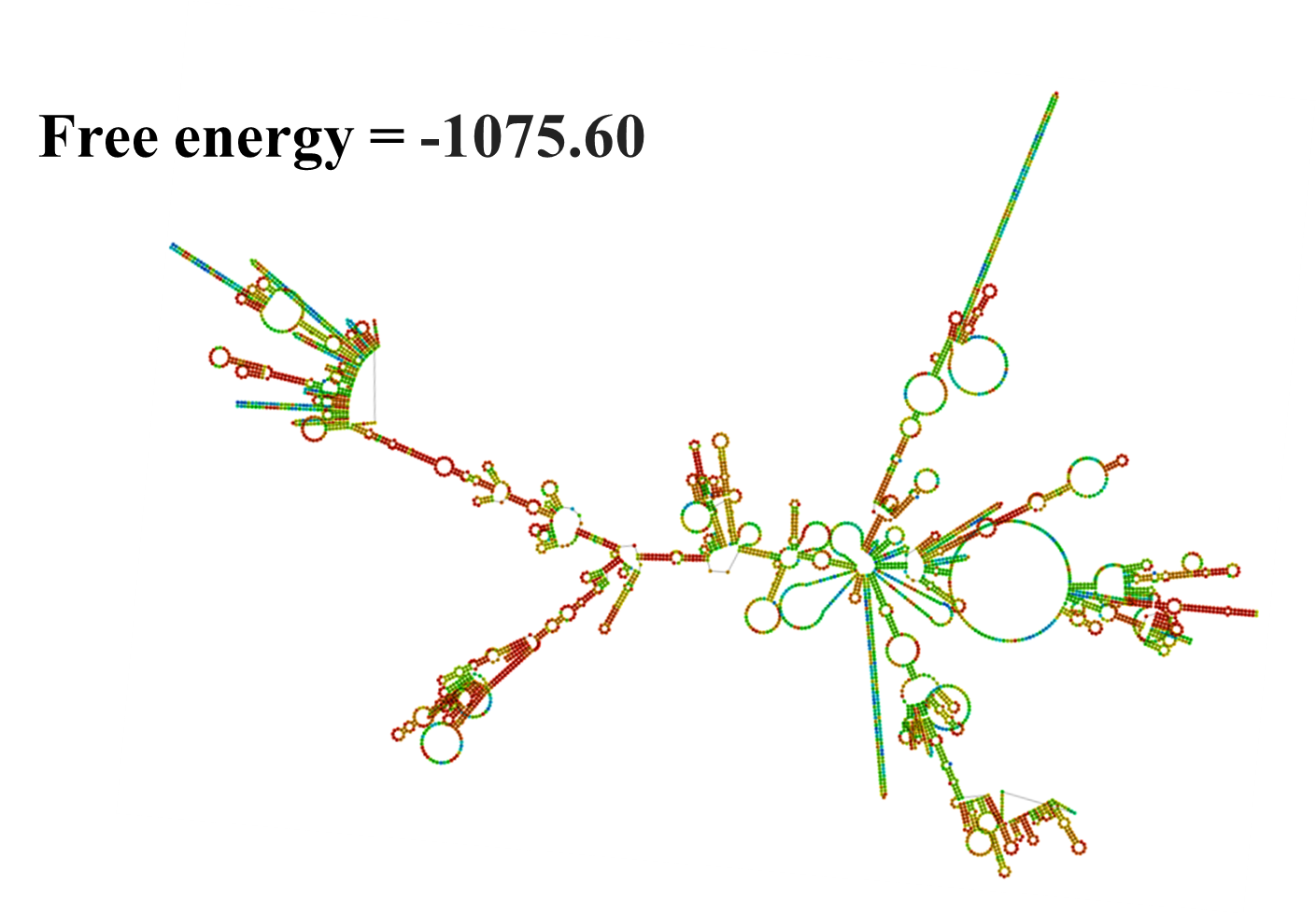

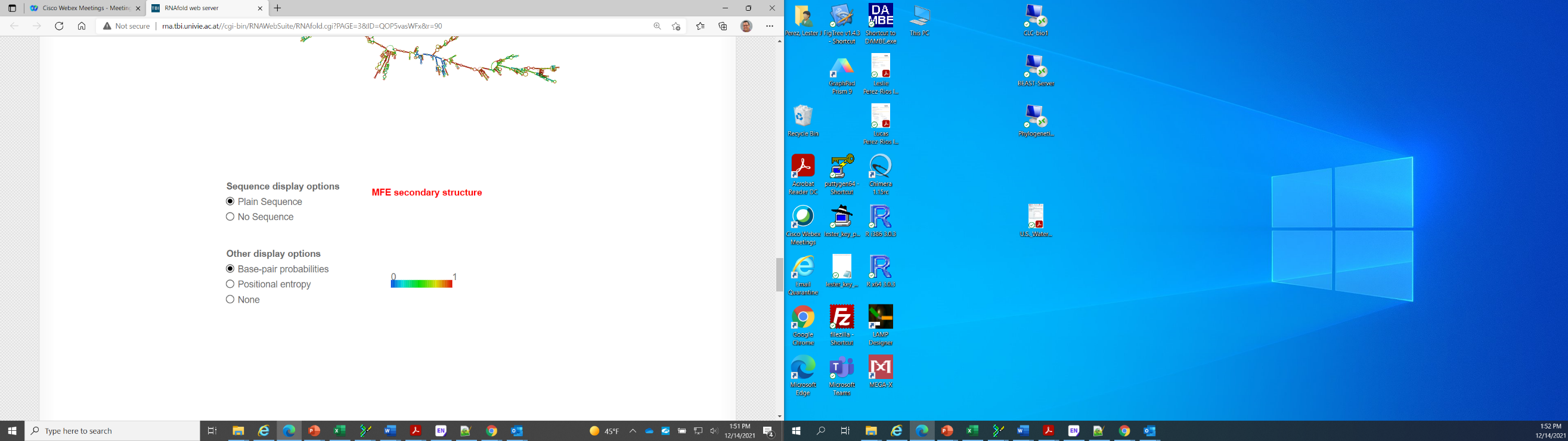


**Figure S5. Comparison of the probability of folding for secondary structures.** A) Secondary structure folding and conservation pattern of pairing for L segment. B) Secondary structure folding and conservation pattern of pairing for M segment. The color scale indicates the level of probability and free energy values for RNA-folding.

**
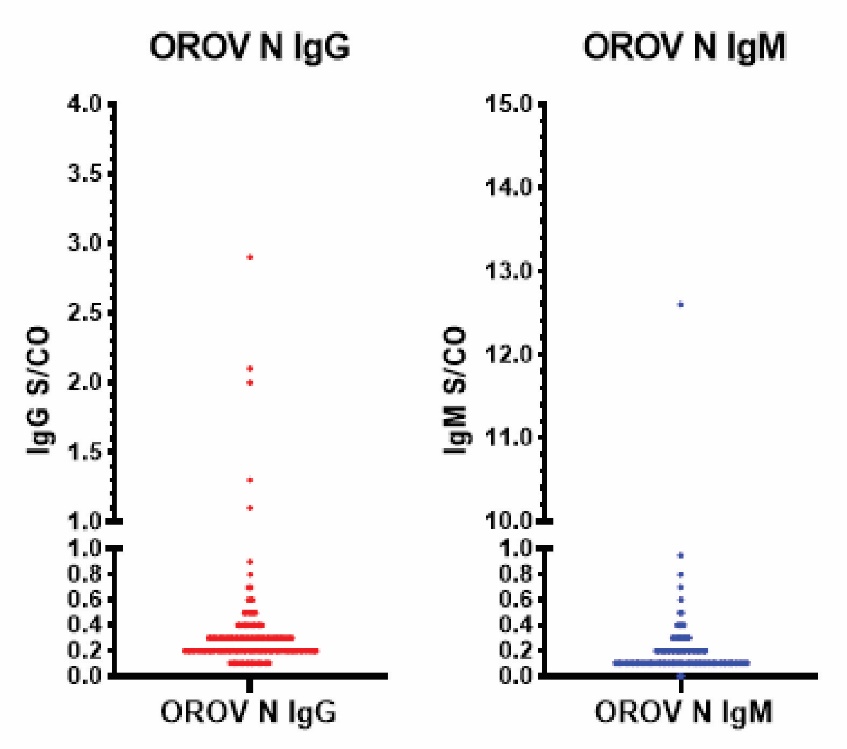
**

**Figure S6. Development of serologic diagnostics for Oropouche.** Specificity of anti-nucleocapsid IgG (*left*) and IgM (*right*) assays tested on a presumed negative cohort of n=500 US donors.


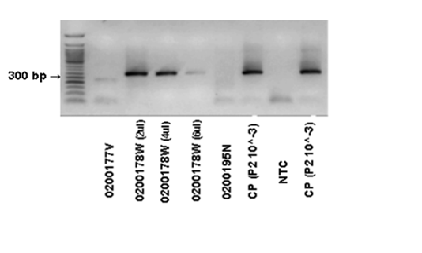


**Figure S7.** **Confirmation of** **Oropouche virus infection in Cucuta.** Oropouche RT-PCR using primers from Lambert & Lanciotti, 2009 et al were used to confirm the presence of (serially diluted) viral RNA in the index patient, 0200178W. A band at 300 nt is of the expected size. Negative samples: 0200177V, 0200195N. Positive controls: 10^3^ virus copies. NTC: non-template control.

**
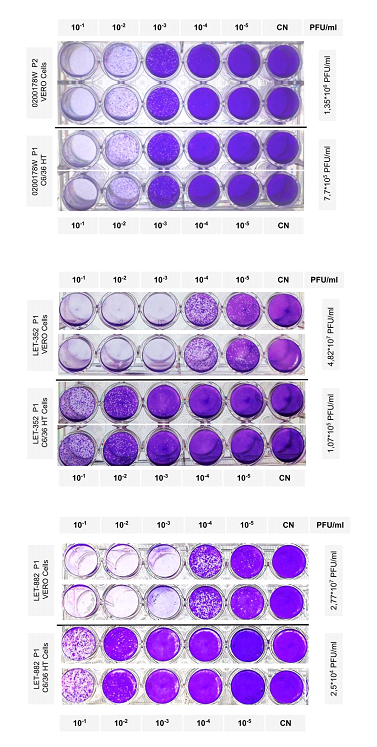
**

**Figure S8. Oropouche virus titration.** Oropouche virus isolated from patient sera (0200178W, LET-352, and LET-882) was serially diluted and used to infect Vero and C6/36 HT cells.

| **Primer/Probe Name** | **Sequence (5′ → 3′)** | **Purpose (Reference)** |
| --- | --- | --- |
| DENV-1, -2, -3 Forward | CAGATCTCTGATGAACAACCAACG | Pan- dengue virus detection (Waggoner et al., 2013) |
| DENV-2 Forward C→T | CAGATCTCTGATGAATAACCAACG |  |
| DENV-3 Forward C→T | CAGATTTCTGATGAACAACCAACG |  |
| DENV-4 Forward | GATCTCTGGAAAAATGAAC |  |
| DENV-1, 3 Reverse | TTTGAGAATCTCTTCGCCAAC |  |
| DENV-2 Reverse | AGTTGACACGCGGTTTCTCT |  |
| DENV-2 Reverse A→G | AGTCGACACGCGGTTTCTCT |  |
| DENV-4 Reverse | AGAATCTCTTCACCAACC |  |
| Probe A | CTCGCGCGTTTCAGCATAT |  |
| Probe B | CTCTCGCGTTTCAGCATAT |  |
| Probe C | CTCTCACGTTTCAGCATATTG |  |
| Probe D | CTCACGCGTTTCAGCATAT |  |
| RNaseP F | AGATTTGGACCTGCGAGCG | Housekeeping gene (Fan et al., 2014) |
| RNaseP R | GAGCGGCTGTCTCCACAAGT |  |
| RNaseP probe | TTCTGACCTGAAGGCTCTGCGCG |  |
| Oropouche Forward | GGCCCATGGTTGACCTTACTTT | Oropouche virus detection (Lambert and Lanciotti 2009) |
| Oropouche Reverse | ACCAAAGGGAAGAAAGTGAAT |  |
| MAYV F | AAGCTCTTCCTCTGCATTGC | Mayaro virus detection (Waggoner et al., 2018) |
| MAYV R1 | TGCTGGAAACGCTCTCTGTA |  |
| MAYV Probe | GCCGAGAGCCCGTTTTTAAAATCAC |  |
| MAYV R2 | TGCTGGAAACGCTCTTTGTA |  |
| CHIK-NS5-F | AAGCTYCGCGTCCTTTACCAAG | Chikungunya virus detection (Gadia et al., 2017) |
| CHIK-NS5-R | CCAAATTGTCCYGGTCTTCCT |  |
| CHIK-NS5-probe | CCAATGTCYTCMGCCTGGACACCTTT |  |
| ZV-E1-F | AARTACACATACCARAACAAAGTGGT | Zika virus detection (Gadia et al., 2017) |
| ZV-E1-R | TCCRCTCCCYCTYTGGTCTTG |  |
| ZV-E-probe | CTYAGACCAGCTGAAR |  |

**Table S1**. Primers and probes used for RT-PCR assays for Arbovirus detection.

**References:**

Waggoner JJ, Rojas A, Mohamed-Hadley A, de Guillén YA, Pinsky BA. Real-time RT-PCR for Mayaro virus detection in plasma and urine. J Clin Virol. 2018 Jan;98:1-4. doi: 10.1016/j.jcv.2017.11.006.

Gadia, C.L.B., Manirakiza, A., Tekpa, G. *et al.* Identification of pathogens for differential diagnosis of fever with jaundice in the Central African Republic: a retrospective assessment, 2008–2010. *BMC Infect Dis* **17,**735 (2017). <https://doi.org/10.1186/s12879-017-2840-8>

Lambert, A.J. and R.S. Lanciotti, Consensus amplification and novel multiplex sequencing method for S segment species identification of 47 viruses of the Orthobunyavirus, Phlebovirus, and Nairovirus genera of the family Bunyaviridae. J Clin Microbiol, 2009. 47(8): p. 2398-404.

Waggoner, J.J., et al., Single-reaction, multiplex, real-time rt-PCR for the detection, quantitation, and serotyping of dengue viruses. PLoS Negl Trop Dis, 2013. 7(4): p. e2116.

Fan J, Cui D, Lau S, Xie G, Guo X, Zheng S, et al. Detection of a novel avian influenza A (H7N9) virus in humans by multiplex one-step real-time RT-PCR assay. BMC Infect Dis [Internet]. 2014 Dec 8;14(1):541. Available from: https://bmcinfectdis.biomedcentral.com/articles/10.1186/1471-2334-14-541

**Table S2.** GenBank Accessions for OROV reference sequences used in phylogenetic trees.

| **Isolate/strain** | **Genbank ID**  **(L segment)** | **Genbank ID**  **(M segment)** | **Genbank ID**  **(S segment)** |
| --- | --- | --- | --- |
| BeAn19991 | KP052850 | KP052851 | KP052852 |
| TRVL9760 | KP026179 | KP026180 | KP026181 |
| TRVL9760 | KP026179 | KC759123 | KP026181 |
| BeH759146 | KP691630 | KP691631 | KP691632 |
| BeAn790177 | KP691627 | NA | KP691629 |
| BeAn789726 | KP691624 | NA | KP691626 |
| BeH759620 | KP691621 | KP691622 | KP691623 |
| BeH759529 | KP691618 | KP691619 | KP691620 |
| BeH759040 | KP691615 | KP691616 | KP691617 |
| BeH759025 | KP691612 | KP691613 | KP691614 |
| BeH759022 | KP691609 | KP691610 | KP691611 |
| BeH759021 | KP691606 | KP691607 | KP691608 |
| BeH759024 | KP691603 | KP691604 | KP691605 |
| NCPV1409261v | MF620129 | MF620128 | NA |
| EC/Esmeraldas/087/2016 | MF926354 | MF926353 | MF926352 |
| BeH543100 | MG747505 | MG747504 | MG747503 |
| BeH389865 | MG747508 | MG747507 | MG747506 |
| BeH390242 | MG747511 | MG747510 | MG747509 |
| BeH472433 | MG747514 | MG747513 | MG747512 |
| BeH472435 | MG747517 | MG747516 | MG747515 |
| BeH421086 | MG747520 | MG747519 | MG747518 |
| BeAn626990 | MG747523 | MG747522 | MG747521 |
| BeAn626990 | MG747523 | MG747522 | AY117135 |
| BeAr19886 | MG747526 | MG747525 | MG747524 |
| BeH29086 | MG747529 | MG747528 | MG747527 |
| BeH29090 | MG747532 | MG747531 | MG747530 |
| BeH121923 | MG747535 | MG747534 | MG747533 |
| BeAr136921 | MG747538 | MG747537 | MG747536 |
| BeAn206119 | MG747541 | MG747540 | MG747539 |

| **Isolate/strain** | **Genbank ID**  **(L segment)** | **Genbank ID**  **(M segment)** | **Genbank ID**  **(S segment)** |
| --- | --- | --- | --- |
| BeAn208402 | MG747544 | MG747543 | MG747542 |
| BeAn208819 | MG747547 | MG747546 | MG747545 |
| BeH355173 | MG747550 | MG747549 | MG747548 |
| BeAr366927 | MG747553 | MG747552 | MG747551 |
| BeH385591 | MG747556 | MG747555 | MG747554 |
| BeH532314 | MG747559 | MG747558 | MG747557 |
| BeH532422 | MG747562 | MG747561 | MG747560 |

**Table S2.** continued.

| BeH532490 | MG747565 | MG747564 | MG747563 |
| --- | --- | --- | --- |
| BeH532500 | MG747568 | MG747567 | MG747566 |
| BeH541140 | MG747571 | MG747570 | MG747569 |
| BeH543629 | MG747574 | MG747573 | MG747572 |
| BeH543760 | MG747577 | MG747576 | MG747575 |
| BeH543857 | MG747580 | MG747579 | MG747578 |
| PPS522H669314 | MG747583 | MG747582 | MG747581 |
| PPS523H669315 | MG747586 | MG747585 | MG747584 |
| PMOH682426 | MG747589 | MG747588 | MG747587 |
| PMOH682431 | MG747592 | MG747591 | MG747590 |
| BeH708139 | MG747595 | MG747594 | MG747593 |
| BeH707287 | MG747598 | MG747597 | MG747596 |
| BeH708717 | MG747601 | MG747600 | MG747599 |
| BeH708717 | MG747601 | MG747600 | HM470138 |
| BeH498913 | MG747604 | MG747603 | MG747602 |
| BeH505768 | MG747607 | MG747606 | MG747605 |
| EC/Esmeraldas/057/2016 | MK506828 | MK506823 | MK506818 |
| EC/Esmeraldas/155/2016 | MK506829 | MK506824 | MK506819 |
| EC/Esmeraldas/171/2016 | MK506830 | MK506825 | MK506820 |
| EC/Esmeraldas/206/2016 | MK506831 | MK506826 | MK506821 |
| EC/Esmeraldas/210/2016 | MK506832 | MK506827 | MK506822 |

| **Isolate/strain** | **Genbank ID**  **(L segment)** | **Genbank ID**  **(M segment)** | **Genbank ID**  **(S segment)** |
| --- | --- | --- | --- |
| OV/Homosapiens/Haiti-1/2014 | MN264267 | NA | NA |
| Bel90435/H853382 | MT879228 | MT879229 | MT879230 |
| NA | AF484424 | NC005775 | AY237111 |
| NA | AF484424 | NC005775 | NC005777 |
| NA | NC005776 | NC005775 | AY237111 |
| NA | NC005776 | NC005775 | NC005777 |
| TRVL-9760 | KC759122 | NA | KC759124 |
| FCT00025/COL/2017 | MK643117 | MK643116 | MK643115 |
| IQT-1690 | KC759125 | NA | KC759127 |
| GML-444479 | KC759128 | NA | KC759130 |
| OV/Homosapiens/Haiti1/2014 | NA | MN264268 | NA |
| IQT1690 | NA | KC759126 | NA |
| GML444479 | NA | KC759129 | NA |
| NCPV1409261 | NA | NA | MF620127 |
| IQT9924 | NA | NA | KF697144 |
| FMD1303 | NA | NA | KF697146 |

**Table S2.** continued

| **Primer/probe** | **Sequence (5'-3')** | **Target** |
| --- | --- | --- |
| *OPV_L2_20F24* | CRATAAACCGRACAATAAATTGGA | L Segment |
| *OPV_L2_115R22* | GTGCTTTTGAACAGAARTGTGA |  |
| *OPV_L2_Probe82U23* | **FAM**-CAAACAAARATTGARCCATGGTC-**BHQ** |  |
| *OPV_M2_6F19* | TGTACTACCAGCAACAAAC | M Segment |
| *OPV_M2_89R19* | CACCAATYTGACTTGTTGA |  |
| *OPV_M2_25L24* | **C**y5-ATWATTARATTCGCCATTGTCAC-**BHQ** |  |

**Table S3.** Primers and probes for RT-qPCR, dual-target Oropouche assay developed in this study.

| **Reagent** | **Volume (µL)** | **Final concentration** | **Source** |
| --- | --- | --- | --- |
| OPV_L2_20F24 | 0.125 | 0.5 µM | IDT Integrated DNA technologies |
| OPV_L2_115R22 | 0.125 | 0.5 µM | IDT Integrated DNA technologies |
| OPV_L2_Probe82U23 | 0.1 | 0.4 µM | IDT Integrated DNA technologies |
| OPV_M2_6F19 | 0.125 | 0.5 µM | IDT Integrated DNA technologies |
| OPV_M2_89R19 | 0.125 | 0.5 µM | IDT Integrated DNA technologies |
| OPV_M2_25L24 | 0.1 | 0.4 µM | IDT Integrated DNA technologies |
| Fwd_Primer_IC^a^ | 0.16 | 1X | Abbott Laboratories |
| Rev_Primer_IC^a^ | 0.18 | 1X | Abbott Laboratories |
| VIC-Probe_IC_BHQ^a^ | 0.13 | 1X | Abbott Laboratories |
| 2X RT‑PCR Buffer AgPath-ID™ | 12.5 | 1X | ThermoFisher Scientific |
| 25X RT‑PCR Enzyme Mix AgPath-ID™ | 1 | 1X | ThermoFisher Scientific |
| Nuclease free water | 5.28 | - | ThermoFisher Scientific |
| Total | **25** |  |  |

**Table S4.** Conditions for RT-qPCR, dual-target Oropouche assay developed in this study.

| **A** | **FAM** | | | | |
| --- | --- | --- | --- | --- | --- |
| Expected | Ct | MR | Final I | Dilution | Positives |
| Ct=32.5 | 33.3 | 1.2 | 1.9 | 10000 | 8/8' |
| Ct=35.5 | 35.8 | 0.9 | 1.3 | 100K | 7/8' |
| Ct=36.5 | 38.5 | 0.7 | 0.5 | 200K | 7/8' |
| Ct=37.5 | 38.7 | 0.7 | 0.4 | 400K | 6/8' |
| Ct=38.5 | 38.1 | 0.8 | 0.6 | 800K | 2/8' |
| Ct=39.5 | 37.6 | 0.7 | 0.8 | 1.6M | 2/8' |
| Ct=40.5 | -1.0 | -1.0 | -1.0 | 3.2M | 0.0 |
|  | **CY5** | | | | |
|  | Ct | MR | Final I | Dilution | Positives |
| Ct=32.5 | 32.2 | 0.5 | 0.2 | 10000 | 8/8' |
| Ct=35.5 | 35.2 | 0.5 | 0.1 | 100K | 8/8' |
| Ct=36.5 | 37.7 | 0.5 | 0.1 | 200K | 7/8' |
| Ct=37.5 | 37.8 | 0.5 | 0.1 | 400K | 3/8' |
| Ct=38.5 | 38.2 | 0.4 | 0.0 | 800K | 2/8' |
| Ct=39.5 | 39.1 | 0.5 | 0.0 | 1.6M | 1/8' |
| Ct=40.5 | 38.7 | 0.5 | 0.0 | 3.2M | 1/8' |

| **B** | **FAM** | | | | |
| --- | --- | --- | --- | --- | --- |
| **Expected** | **Ct** | **MR** | **Final I** | **Dilution** | **Positives** |
| Ct=33.9 | 36 | 1.1 | 3 | 100K | 8/8' |
| Ct=34.9 | 37.4 | 1.9 | 2.9 | 200K | 8/8' |
| Ct=35.9 | 38.4 | 0.9 | 2.7 | 400K | 2/8' |
| Ct=36.9 | 38.8 | 0.8 | 2.6 | 800K | 5/8' |
| Ct=37.9 | -1.0 | -1.0 | -1.0 | 1.6M | 0/8' |
| Ct=38.9 | 38.9 | 0.8 | 2.5 | 3.2M | 1/8' |
| Ct=39.9 | -1.0 | -1.0 | -1.0 | 6.4M | 0/8' |
|  | 37.3 | 0.8 | 6.1 | N/A | 1/8' |
|  | **CY5** | | | | |
| **Expected** | **Ct** | **MR** | **Final I** | **Dilution** | **Positives** |
| Ct=33.9 | 36 | 0.5 | 0 | 100K | 8/8' |
| Ct=34.9 | 37.7 | 0.5 | 0.2 | 200K | 6/8' |
| Ct=35.9 | 37.5 | 0.5 | 0.2 | 400K | 6/8' |
| Ct=36.9 | 38.0 | 0.5 | 0.1 | 800K | 1/8' |
| Ct=37.9 | 38.0 | 0.5 | 0.2 | 1.6M | 3/8' |
| Ct=38.9 | 38.6 | 0.5 | 0.2 | 3.2M | 1/8' |
| Ct=39.9 | -1.0 | -1.0 | -1.0 | 6.4M | 0/8' |
|  | 40.3 | 0.3 | 0.1 | N/A | 1/8' |

**Table S5.** RT-qPCR specificity and drop out evaluations with OROV virus lysate dilutions. A) Vero CCL81 cell lysate diluted in human serum. B) C6/36 cell lysate diluted in human serum

| **Total Samples** | **Sample Number** | **Sample ID** | **FAM** | **Cy5** |
| --- | --- | --- | --- | --- |
| 1 | 7 | 0200175T | 33.67 | 34.02 |
| 2 | 8 | 0200178W | 20.67 | 23.23 |
| 3 | 82 | ISA-BRI-1445O | 33.69 | -1 |
| 4 | 87 | ISA-BRI-0540T | 34.79 | -1 |
| 5 | 158 | 0300243I | 34.13 | -1 |
| 6 | 166 | 0300186D | 31.04 | 32.98 |
| 7 | 239 | 0300333U | 34.82 | -1 |
| 8 | 240 | 0300325M | 34.94 | -1 |
| 9 | 247 | 0300326N | 32.45 | -1 |
| 10 | 251 | 0300271K | 33.84 | -1 |
| 11 | 252 | 0300343E | 34.68 | -1 |
| 12 | 253 | 0300335W | 34.7 | -1 |
| 13 | 254 | 0300224P | 34.72 | -1 |
| 14 | 256 | 0300288B | 34.83 | -1 |
| 15 | 257 | 0300280T | 33.98 | -1 |
| 16 | 258 | 0300272L | 34.38 | -1 |
| 17 | 259 | 0300344F | 34.74 | -1 |
| 18 | 265 | 0300282V | 34.84 | -1 |
| 19 | 266 | 0300242H | 34.8 | -1 |
| 20 | 267 | 0300314B | 33.73 | -1 |
| 21 | 269 | 0300298L | 34.26 | -1 |
| 22 | 270 | 0300290D | 33.73 | -1 |
| 23 | 271 | 0300291E | 33.29 | -1 |
| 24 | 272 | 0300251Q | 34.48 | -1 |
| 25 | 275 | 0300307U | 34.57 | -1 |
| 26 | 276 | 0300299M | 34.56 | -1 |
| 27 | 279 | 0300332T | 34.59 | -1 |
| 28 | 282 | 0300308V | 33.79 | -1 |
| 29 | 628 | 0300101W | 37.69 | -1 |
| 30 | 637 | 0300087I | 37.27 | 36.27 |
| 31 | 643 | 0100109F | -1 | 38.95 |
| 32 | 660 | LET-175 | 36.33 | -1 |
| 33 | 663 | 0100090L | 36.87 | -1 |
| 34 | 698 | 0200119P | 38.03 | -1 |
| 35 | 724 | LET-110 | 38.18 | -1 |
| 36 | 736 | LET-138 | 36.35 | -1 |
| 37 | 737 | LET-130 | 37.31 | -1 |
| 38 | 738 | LET-131 | 36.22 | 39.2 |

| **Total Samples** | **Sample Number** | **Sample ID** | **FAM** | **Cy5** |
| --- | --- | --- | --- | --- |
| 39 | 739 | LET-123 | 37.66 | -1 |
| 40 | 740 | LET-115 | 37.68 | -1 |
| 41 | 742 | LET-68 | 37.24 | -1 |
| 42 | 743 | LET-69 | 37.2 | -1 |
| 43 | 744 | LET-140 | 36.9 | -1 |
| 44 | 745 | LET-132 | 36.22 | 39.54 |
| 45 | 746 | LET-124 | 36.68 | 37.7 |
| 46 | 748 | LET-76 | 37.09 | -1 |
| 47 | 751 | LET-141 | 35.38 | -1 |
| 48 | 752 | LET-133 | 34.64 | -1 |
| 49 | 753 | LET-125 | 37.18 | -1 |
| 50 | 756 | LET-78 | 35.86 | 37.89 |
| 51 | 757 | LET-70 | 37.29 | -1 |
| 52 | 763 | LET-79 | 35.02 | -1 |
| 53 | 765 | LET-143 | 37.31 | -1 |
| 54 | 767 | LET-104 | 37.21 | -1 |
| 55 | 768 | LET-96 | 37.39 | -1 |
| 56 | 770 | LET-80 | 37.2 | -1 |
| 57 | 771 | LET-72 | 37.1 | -1 |
| 58 | 775 | LET-97 | 35.58 | -1 |
| 59 | 776 | LET-89 | 37.2 | -1 |
| 60 | 777 | LET-81 | 37.1 | -1 |
| 61 | 778 | LET-121 | 36.07 | -1 |
| 62 | 781 | LET-106 | 38.29 | 38.4 |
| 63 | 782 | LET-98 | 37.14 | 38.45 |
| 64 | 786 | LET-31 | 36.75 | -1 |
| 65 | 787 | LET-43 | 35.89 | -1 |
| 66 | 791 | LET-42 | 37.3 | -1 |
| 67 | 799 | LET-178 | 36.36 | -1 |
| 68 | 803 | LET-187 | 35.44 | -1 |
| 2 | 60 | ISA-BRI-1084 | -1 | 37.19 |
| 3 | 89 | ISA-BRI-0917G | 38.19 | -1 |
| 4 | 187 | 0300255U | -1 | 38.83 |
| 5 | 273 | 0300323K | 38.37 | -1 |
| 6 | 456 | 0300572Z | 37.18 | -1 |
| 7 | 461 | 0300532L | 37.53 | -1 |

| **Total Samples** | **Sample Number** | **Sample ID** | **FAM** | **Cy5** |
| --- | --- | --- | --- | --- |
| 8 | 466 | 300628 | 39.17 | -1 |
| 9 | 516 | 0300537Q | 37.16 | -1 |
| 10 | 543 | 0300540T | 37.06 | -1 |
| 11 | 552 | 0300108D | 34.24 | -1 |
| 12 | 567 | 0300025Y | 35.23 | -1 |
| 13 | 579 | 0300048V | 37.13 | -1 |
| 14 | 598 | 0300598 | 37.5 | -1 |
| 15 | 633 | 0300623 | 37.35 | -1 |
| 16 | 648 | LET-157 | 38.15 | -1 |
| 17 | 662 | 0100051Y | 38.17 | -1 |
| 18 | 671 | LET-174 | 38.21 | -1 |
| 19 | 682 | LET-173 | 37.24 | -1 |
| 20 | 702 | LET-58 | 37.22 | -1 |
| 21 | 705 | LET-82 | 38.15 | -1 |
| 23 | 760 | LET-94 | 38.22 | -1 |
| 27 | 801 | LET-55 | 38.17 | -1 |
| 28 | 813 | LET-51 | 38.43 | -1 |

**Table S6. Cycle threshold (Ct) values for Oropouche qPCR positive cases.**

**Table S7. Classification of samples for Oropouche serology positive cases.**

| **Samples tested IGG positive** | | |
| --- | --- | --- |
| 0300362 | 0300579 | LET-154 |
| 0300375 | 0300583 | LET-155 |
| 0300453 | 0300584 | LET-163 |
| 0300463 | 0300587 | LET-170 |
| 0300464 | 0300593 | LET-177 |
| 0300485 | 0300596 | LET-182 |
| 0300513 | LET-114 | LET-183 |
| 0300549 | LET-116 | LET-59 |
| 0300560 | LET-127 | LET-60 |
| 0300568 | LET-136 | LET-65 |
| 0300576 | LET-145 | LET-95 |
| **Samples tested IGM positive** | | |
| 0300462 | LET-101 | LET-76 |
| 0300501 | LET-160 | LET-89 |
| 0300618 | LET-197 |  |
| **Samples tested IGM and IGG positive** | | |
| 0300478 | LET-110 | LET-70 |
| LET-103 | LET-123 | LET-96 |
| LET-105 | LET-132 | LET-98 |
| **Samples tested IGG and PCR positive** | | |
| 0300242 | LET-121 | LET-178 |
| 0300290 | LET-138 | LET-55 |
| 0300343 | LET-157 | LET-80 |
| 0300537 | LET-173 | LET-81 |
| LET-104 | LET-175 | LET-82 |
| **Samples tested IGM and PCR positive** | | |
| 0300288 | LET-76 | LET-89 |
| **Samples tested IGM, IGG and PCR positive** | | |
| LET-110 | LET-132 | LET-96 |
| LET-123 | LET-70 | LET-98 |

| **Characteristic** | **Dengue cases** | | **Oropouche cases** | |
| --- | --- | --- | --- | --- |
|  | **Odds Ratio** | **Confidence Intervals 95%** | **Odds Ratio** | **Confidence Intervals 95%** |
| Age (years) | 0.79 | 0.73-0.85 | 1.18 | 1.0-1.36 |
| Sex | 0.05 | 0.87-1.25 | 1.1 | 0.72-1.64 |
| Location of sample collection | 0.99 | 0.88-1.11 | 2.98 | 2.03-4.46 |
| Year of sample collection | 0.58 | 0.51-0.66 | 1.75 | 1.1-3.08 |
| Climate phase | 1.61 | 1.37-1.90 | 1.64 | 1.16-2.31 |
| Headache | 0.64 | 0.45-0.94 | 0.43 | 0.22-0.92 |
| Muscle pain | 0.54 | 0.41-0.71 | 0.71 | 0.35-1.60 |
| Odynophagia | 0.79 | 0.64-0.98 | 2.32 | 1.42-3.71 |
| Retroorbital pain | 0.82 | 0.68-0.99 | 0.72 | 0.47-1.12 |
| Red eyes | 1.15 | 0.93-1.43 | 1.72 | 1.1-2.74 |
| Chills | 0.88 | 0.70-1.10 | 0.67 | 0.41-1.15 |
| Abdominal pain | 1.42 | 0.19-1.70 | 0.98 | 0.62-1.51 |
| Weakness | 1.28 | 1.1-1.54 | 1.31 | 0.84-2.10 |
| Skin Rash | 3.25 | 2.56-4.13 | 0.8 | 0.41-1.44 |
| Dizziness | 0.9 | 0.75-1.07 | 1.16 | 0.75-1.81 |
| Vomit | 1.1 | 0.93-1.32 | 0.6 | 0.39-0.91 |
| Petechiae | 12.49 | 5.25-34.41 | 0 | - |
| Diarrhea | 1.14 | 0.92-1.42 | 0.94 | 0.52-1.63 |

**Table S8. Bivariate analysis using logistic regression.** Red indicates significant increase in the odds while blue indicates reduction.

| **Characteristic** | **Model estimate** | **P value** | **Odds Ratio** | **Confidence Intervals 95%** |
| --- | --- | --- | --- | --- |
| Intercept | -0.7 | <0.01 | 0.5 | 0.35-0.73 |
| Age (years) | -0.2 | <0.01 | 0.85 | 0.78-0.91 |
| Year of sample collection | -0.2 | <0.05 | 0.83 | 0.71-0.98 |
| Muscle pain | -0.4 | <0.05 | 0.7 | 0.51-0.94 |
| Weakness | 0.3 | <0.01 | 1.29 | 1.1-1.55 |
| Skin Rash | 0.8 | <0.01 | 2.33 | 1.75-3.09 |
| Petechiae | 1.3 | <0.01 | 3.61 | 1.38-9.47 |

**Table S9.** Final logistic regression model for dengue cases with estimation of robust variance after selection of best model based on AIC. Climate, spatial-temporal, demographic and clinical variables.

| **Characteristic** | **Model estimate** | **P value** | **Odds Ratio** | **Confidence Intervals 95%** |
| --- | --- | --- | --- | --- |
| Intercept | -4.4 | <0.01 | 0.01 | 0.00-0.03 |
| Location of sample collection | 1.0 | <0.01 | 2.63 | 1.82-3.80 |
| Climate phase | 0.5 | <0.05 | 1.65 | 1.1-2.49 |
| Odynophagia | 0.7 | <0.05 | 2.03 | 1.22-3.39 |

**Table S10.** Final logistic regression model for Oropouche cases with estimation of robust variance after selection of best model based on AIC. Climate, spatial-temporal, demographic and clinical variables.

| **Characteristic** | | **Model estimate** | **P value** | **Odds Ratio** | **Confidence Intervals 95%** |
| --- | --- | --- | --- | --- | --- |
| Intercept | | -1.8 | <0.01 | 0.2 | 0.1-0.3 |
| Age (years) | 1 | 1.2 | <0.01 | 3.2 | 2.0-5.2 |
|  | 2 | -0.0 | 0.9 | 1.0 | 0.6-1.6 |
|  | 3 | 0.0 | 0.9 | 1.0 | 0.6-1.6 |
|  | 4 | 0.2 | 0.9 | 1.0 | 0.6-1.3 |
|  | 5 | -0.2 | 0.3 | 0.8 | 0.4-1.3 |
| Odynophagia | | -0.4 | <0.01 | 0.7 | 0.5-0.9 |
| Red eyes | | 0.5 | <0.01 | 1.6 | 1.2-2.1 |
| Abdominal pain | | 0.2 | 0.07 | 1.2 | 1.0-1.4 |
| Weakness | | 0.2 | <0.05 | 1.2 | 1.0-1.5 |
| Skin rash | | 0.8 | <0.01 | 2.2 | 1.7-2.9 |
| Petechiae | | 1.2 | <0.01 | 3.5 | 1.3-9.2 |

**Table S11.** Final logistic regression model for dengue cases with estimation of robust variance after selection of best model based on AIC. Demographic and clinical variables only.

| **Characteristic** | **Model estimate** | **P value** | **Odds Ratio** | **Confidence Intervals 95%** |
| --- | --- | --- | --- | --- |
| Intercept | 1.8 | <0.01 | 5.9 | 3.9-8.6 |
| Odynophagia | 1.2 | <0.01 | 3.2 | 2.4-4.3 |
| Abdominal pain | 0.9 | <0.01 | 2.5 | 2.0-3.0 |
| Weakness | -0.5 | <0.01 | 0.6 | 0.5-0.7 |
| Dizziness | -0.7 | <0.01 | 0.5 | 0.4-0.6 |
| Sex-Female | 0.3 | <0.01 | 1.3 | 1.1-1.6 |
| Skin rash | -0.7 | <0.01 | 0.5 | 0.4-0.7 |
| Retroorbital pain | -0.2 | 0.1 | 0.8 | 0.7-1.0 |
| Muscle pain | -0.4 | <0.05 | 0.7 | 0.5-1.0 |
| Red eyes | -0.4 | <0.05 | 0.7 | 0.5-1.0 |

**Table S12.** Final logistic regression model for Oropouche cases with estimation of robust variance after selection of best model based on AIC. Demographic and clinical variables only.
